# Scalable and Sustainable Chitosan/Carbon Nanotubes Composite Protective Layer for Dendrite-Free and Long-Cycling Aqueous Zinc-Metal Batteries

**DOI:** 10.1007/s40820-025-01837-7

**Published:** 2025-07-08

**Authors:** Jinchang Wang, Alessandro Innocenti, Hang Wei, Yuanyuan Zhang, Jingsong Peng, Yuanting Qiao, Weifeng Huang, Jian Liu

**Affiliations:** 1 College of Chemistry and Chemical Engineering, Inner Mongolia Key Laboratory of Rare Earth Catalysis, College of Energy Material and Chemistry, Institute for Green Chemistry and Environmental Science, Inner Mongolia University, Hohhot, 010021 Inner Mongolia People’s Republic of China; 2 Zentrum für Sonnenenergie- und Wasserstoff-Forschung Baden-Württemberg, 89081 Ulm, Germany; 3 China-Italy Joint Laboratory of In-Situ/Operando Instrumentation Beijing Science Star Technology Co. Ltd., Beijing, People’s Republic of China; 4 Department of Chemical Engineering, Faculty of Engineering, Swansea University, Swansea, SA1 8EN UK; 5 DICP-Surrey Joint Centre for Future Materials, Department of Chemical and Process Engineering and Advanced Technology Institute, University of Surrey, Guildford, Surrey GU2 7XH UK

**Keywords:** Zn anode, Chitosan/CNTs, Protective layer, Techno-economic analysis, Biomimetic

## Abstract

Rechargeable aqueous zinc (Zn)-metal batteries hold great promise for next-generation energy storage systems. However, their practical application is hindered by several challenges, including dendrite formation, corrosion, and the competing hydrogen evolution reaction. To address these issues, we designed and fabricated a composite protective layer for Zn anodes by integrating carbon nanotubes (CNTs) with chitosan through a simple and scalable scraping process. The CNTs ensure uniform electric field distribution due to their high electrical conductivity, while protonated chitosan regulates ion transport and suppresses dendrite formation at the anode interface. The chitosan/CNTs composite layer also facilitates smooth Zn^2+^ deposition, enhancing the stability and reversibility of the Zn anode. As a result, the chitosan/CNTs @ Zn anode demonstrates exceptional cycling stability, achieving over 3000 h of plating/stripping with minimal degradation. When paired with a V_2_O_5_ cathode, the composite-protected anode significantly improves the cycle stability and energy density of the full cell. Techno-economic analysis confirms that batteries incorporating the chitosan/CNTs protective layer outperform those with bare Zn anodes in terms of energy density and overall performance under optimized conditions. This work provides a scalable and sustainable strategy to overcome the critical challenges of aqueous Zn-metal batteries, paving the way for their practical application in next-generation energy storage systems.

**Supplementary Information:**

The online version contains supplementary material available at 10.1007/s40820-025-01837-7.

## Highlights

- A sustainable protective layer for Zn anodes integrating carbon nanotubes (CNTs) and chitosan via a simple scraping process to inhibit dendrite growth and side reactions.
- Chitosan’s polar functional groups enhance Zn^2+^ transport, and the even distribution of CNTs lowers local current density, enabling a uniform electric field to regulate Zn deposition.
- Benefiting from the chitosan/CNTs protective layer, the Zn//Zn and Zn//V_2_O_5_ cells showcase significantly enhanced electrochemical performance, and technical economic analyses demonstrate their practical applications.

**Supplementary Information:**

The online version contains supplementary material available at 10.1007/s40820-025-01837-7.

## Graphical abstract

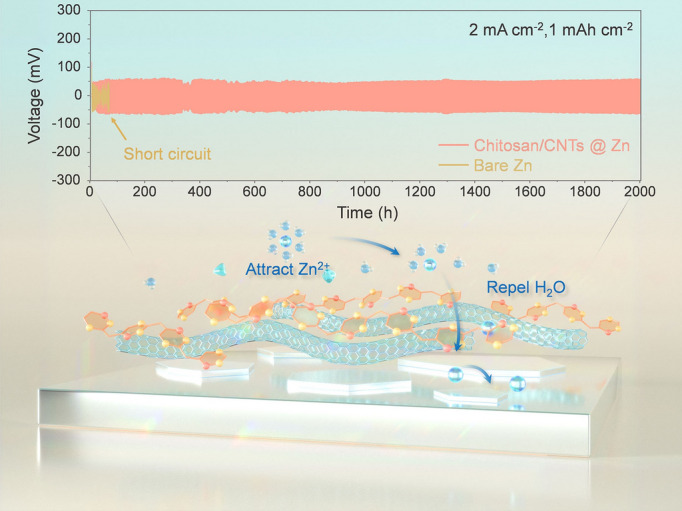

## Introduction

Lithium-ion and lithium-metal batteries, along with other organic system batteries [1–3], are vital for the green energy industry. However, their application in emerging grid-scale energy storage systems is hindered by lithium scarcity and safety issues. This drives researchers to develop new energy storage battery systems with intrinsic safety, environmental-friendliness and low cost. Aqueous zinc-ion batteries are emerging as promising candidates for next-generation large-scale energy storage due to their safety features, environmental compatibility, and cost-effectiveness [4, 5]. The use of aqueous electrolytes in these batteries enhances safety, improves ionic conductivity, and reduces costs [6, 7]. However, it still remains some critical challenges such as corrosion and HER arising from the thermodynamic incompatibility between H_2_O and zinc for practical applications [8–10]. Additionally, zinc dendrite formation during plating and stripping processes poses a significant barrier to the practical use of these batteries, as it might lead to short-circuits [11].

To work out these issues, zinc anodes are usually modified through artificial protective layers [12, 13]. Typically inorganic materials [14–18], alloys [19–22], carbon-based materials [23–26], or polymers [27–30], function by physically isolating metallic zinc from aqueous electrolytes while regulating ion flux. This dual mechanism effectively can indeed suppress parasitic HER and enhances coulombic efficiency. However, conventional single-component protective layers show limited efficacy in dendrite suppression and long-term cycling stability, particularly under practical operating conditions.

The emerging composite protective layer strategy can address these limitations by combining complementary materials. Especially, carbon nanotubes (CNTs) show promise due to their high electrical conductivity and large specific surface area, which synergistically reduce Zn^2+^ nucleation barriers. For example, β–CD-grafted CNTs films [31] and CNTs-modified Zn_2_SiO_4_ nanospheres [32] can facilitate uniform deposition of Zn^2^⁺ and suppress H_2_O-caused side reactions. However, these protective layers often require polyvinylidene difluoride (PVDF) as a binder. The hydrophobic nature of PVDF can increase interfacial impedance and inadequate ion transport. Although powder-metallurgy-based [33] and electrodeposition strategies [34] can incorporate CNTs into zinc anodes without binders, these methods are complex and energy-consuming. Moreover, CNTs recycling is often overlooked. Thus, achieving efficient and recyclable CNTs utilization in zinc anodes remains challenging. As a natural polymer, chitosan possesses several advantages, such as cost-effective, abundant H-bonded groups and strong coordination ability. Its abundant hydrogen bonds endow it with excellent self-adhesive properties. This allows chitosan to bind effectively with CNTs without extra binders. More importantly, chitosan’s good solubility in acetic acid makes the recycling of CNTs feasible.

Inspired by plant cell wall, we develop a sustainable chitosan/CNTs composite protective layer on the zinc anode via a simple and energy-efficient scraping coating method. The chitosan in the composite like a soft matrix not only serves as a binder, but also functions as Lewis basic sites, effectively attracting and capturing Zn^2+^, thereby regulating Zn^2+^ transport. Moreover, the CNTs act like the fibrous component, providing strength and conductivity, and the abundant CNTs within the protective layer facilitate the redistribution of charge at the anode interface, which reduces the local current density, resulting in a lower nucleation overpotential for zinc and effectively inhibiting dendrite growth. Accordingly, the Zn//Zn symmetric batteries can maintain stable shelf-recovery performances for over 3000 h at a current density of 1 mA cm^−2^. And the reversibility of chitosan as a binder enables the successful recovery of high-value CNTs, demonstrating a nice sustainability. This study provides a novel design idea for highly efficient use of electrodes.

## Experimental and Calculation

### Preparation of Materials

#### Chitosan/CNTs Protective Layer Preparation

Three hundred milligram of chitosan were added into 7.5 mL of glacial acetic acid (3 wt%) and stirred for 1 h to fully dissolve the chitosan to form a viscous gel. Hundred milligram CNTs were added and strongly stirred at 1500 r min^−1^ for 3 h to disperse them. The dispersion was ultrasonicated for 3 h after standing 0.5 h for defoaming. The above slurry was scraped onto the zinc foil using a scraper and dried at room temperature.

#### Re-extraction of CNTs

After the cycling, the battery was disassembled, and the chitosan/CNTs protective layers were carefully peeled off. Excess glacial acetic acid was added, and the solution was thoroughly stirred to fully dissolve the layers. After centrifugation (10,000 r min^−1^, 30 min), the precipitate was washed with a large amount of deionized water and centrifuged again. The sediment was collected and dried.

### Battery Assembly

#### Assembly of Zn//Zn Symmetric Batteries and Zn//V_2_O_5_ Batteries

The symmetrical battery was assembled using CR2023 coin cells with chitosan/CNTs @ Zn and bare Zn electrode as anode and cathode. 2 M Zn(CF_3_SO_3_)_2_ as the electrolyte and glass fiber was applied as the separator. For the preparation of the cathode: commercial V_2_O_5_ was mixed with super P and PVDF at a mass ratio of 7:2:1 in NMP, then coated on carbon paper and dried at 80 °C for 12 h. The mass loading of active material was ~ 1.0 mg cm^−2^. The chitosan/CNTs @ Zn//V_2_O_5_ (bare Zn//V_2_O_5_) full battery was prepared by dropping 2 M Zn (CF_3_SO_3_)_2_ on a glass fiber separator in normal atmosphere using commercial V_2_O_5_ as cathode and chitosan/CNTs @ Zn (bare Zn) as anode.

#### Assembly of Zn//Cu Asymmetric Batteries

Chitosan/CNTs @ Zn//Cu and Zn//Cu asymmetric batteries were assembled with chitosan/CNTs @ Zn (bare Zn) as the anode, Cu foil as the cathode, and the electrolyte was 2 M Zn (CF_3_SO_3_)_2_. The coulombic efficiency of zinc plating/stripping was measured using asymmetric batteries with a current of 1 mA cm^−2^ and a charging cutoff voltage of 1 V.

#### Assembly of Al//Al Symmetric Batteries

The Al//Al symmetric battery was assembled with the bare Al or chitosan/CNTs @ Al anode. Regarding the electrolyte, in the glove box (< 0.1 ppm water and oxygen) in argon atmosphere, anhydrous aluminum chloride with a molar ratio of 1:1.3 was slowly added to dry 1-ethyl-3-methylimidazolium chloride ([EMIm] Cl), stirring under argon protection for 2 h. Then, 60 µL were added to a glass fiber separator dropwise.

### Characterization

The samples were characterized using various techniques. X-ray diffraction (XRD) was performed with a Bruker D8 Advance diffractometer using Cu Kα radiation (*λ* = 1.5406 Å) to identify the samples. The microstructure and size of the prepared samples were observed using a Hitachi S-4800 scanning electron microscope (SEM). X-ray photoelectron spectroscopy (XPS) was conducted on a Thermo Scientific ESCALAB Xi + using Al Kα as the excitation source, while the CNTs were observed using a TEM (HT 7800). Fourier transform infrared spectroscopy (FT-IR) was used to analyze chitosan, utilizing a Thermo Fisher iS50 spectrometer. The zeta potential of the electrodes was measured with a 90PLUS particle size analyzer.

For in situ computed tomography (CT) experiments, a photon energy of 40 kV was used, with an object-to-detector distance of 15 mm. The detection system included a 20-μm YAG-Ce scintillator, an optical microscope (magnification range 1–40), and a low-noise fast-readout CCD camera with 2048 × 2048 pixels and 16-bit dynamic range, and data were collected using DEEP-Insight spectroscopy provided by Beijing Science Star Technology Co., LTD. Three-dimensional optical images were obtained using a VHX-6000 system. Lastly, Raman intensity change maps during Zn deposition were recorded using in situ Raman spectroscopy (DEEP-INRS-II, Beijing Science Star Technology Co., LTD.), with a laser power of 5 mW, an integration time of 10 s, an acquisition interval of 30 s, and a 20 × objective lens.

## Results and Discussion

### Preparation and Characterization of Chitosan/CNTs Protective Layer

**Fig. 1:**
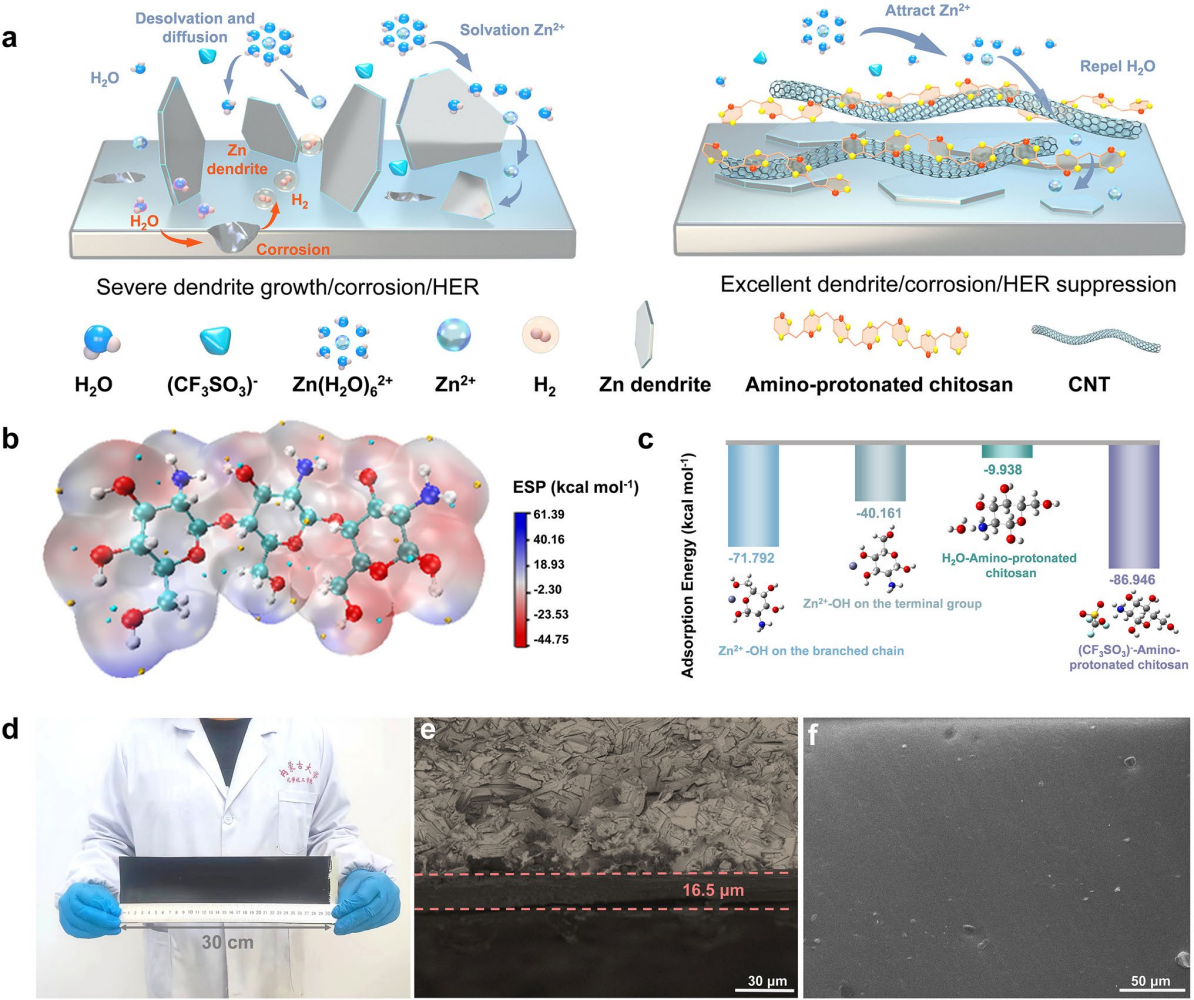
Composition and morphology analysis of chitosan/CNTs protective layer. **a** Schematic illustration of the stabilization of Zn anode with chitosan/CNTs protective layer. **b** ESP of chitosan molecule. **c** Adsorption energy between different molecules. **d** Photo of chitosan/CNTs protective layer. **e** Cross-sectional SEM image of chitosan/CNTs @ Zn and **f** surface SEM image

On bare Zn, hydrated Zn^2+^ undergo desolvation and diffusion, during which H_2_O inevitably attack the zinc surface, leading to severe corrosion and HER. Additionally, slow interfacial kinetics contribute to the subsequent growth of zinc dendrites during deposition [35, 36]. It has been proved that CNTs has a high specific surface area and can directly alter the electron distribution on the zinc anode, thereby facilitating the uniform deposition of zinc [37]. However, the irreversibility of the reported traditional binders impedes the recycling of high-value CNTs. If the binder is recyclable nature resins, then the CNTs may be highly recycling. Furthermore, if this binder has protonated groups, it should also enable to effectively attract and capture of H_2_O as well as (CF_3_SO_3_)⁻, promoting the uniform deposition of Zn^2+^, as shown in Fig. 1a.

The electrostatic potential analysis (ESP) indicates a significant electron cloud density of − 44.75 kcal mol^−1^ at the hydroxyl groups in chitosan (Fig. 1b). These electron-rich hydroxyl sites exhibit strong electrostatic interactions with Zn^2+^, facilitating the rapid desolvation of hydrated Zn^2+^ and providing sites for Zn^2+^ transport [38]. This interaction effectively prevents the undesirable interactions between anions, H_2_O, and metallic zinc, thereby reducing side reactions. The lowest unoccupied molecular orbital (LUMO) and highest occupied molecular orbital (HOMO) energy levels for chitosan and H_2_O, revealed that chitosan has a narrower energy gap compared to H_2_O (Fig. S1), indicating its superior electron transport capacity. Furthermore, the lower LUMO energy of chitosan suggests enhanced adsorption on the zinc surface relative to H_2_O, thereby inhibiting the HER and stabilizing the zinc anode [39].

Building on these insights, we chose natural chitosan as the sustainable binder for CNTs, given its natural abundance, biodegradability, and functional groups (–NH₂, –OH) that facilitate ion regulation. When chitosan was dissolved in an acidic solution, such as glacial acetic acid, protonation of the amino groups occurred, forming positively charged cationic groups [40], as shown in Fig. S2. In the FT-IR spectrum, the 1590 cm^−1^ peak corresponded to the N–H bending vibration in chitosan powder (Fig. S3). The introduction of H^+^ from glacial acetic acid caused a red shift in the N–H bending vibrations in both the chitosan film and the chitosan/CNTs film, suggesting the protonation of the amino groups to form –NH_3_^+^ [41]. Moreover, the 1060 cm^−1^ peak in chitosan disappeared with the introduction of glacial acetic acid, while new peaks appeared at 1080 and 1050 cm^−1^, indicating that the structure of chitosan changes during film formation in the acidic environment [42]. In solution, chitosan acts as a positively charged polyelectrolyte with significant adsorption capacity, enabling direct film formation on zinc surfaces. Upon adding chitosan to acetic acid, the zeta potential increased from + 5.58 to + 39.35 mV (Fig. S4), which provided strong evidence for the generation of protonated amino groups. Subsequent XPS analysis confirmed the presence of an -NH_3_^+^ peak (Fig. S5), further supporting amino group protonation. These protonated amino groups, with their positive charge, are key to adsorbing (CF_3_SO_3_)⁻ and H_2_O on the electrode surface.

Using DFT calculations (Fig. 1c), we quantify the adsorption energy of protonated amino groups with (CF_3_SO_3_)⁻ and H_2_O. The adsorption energy between protonated amino groups and H_2_O is − 9.938 kcal mol^−1^, while for (CF_3_SO_3_)⁻, it is − 86.946 kcal mol^−1^. Negative adsorption energy implies that the protonated amino groups have an affinity for (CF_3_SO_3_)⁻ and H_2_O, leading to effective adsorption and stabilization of these species on the surface, reducing corrosion and zinc dendrite formation. This anchoring also promotes the smooth diffusion of Zn^2+^ and the uniform distribution of interface ions. In addition, the hydroxyl groups at the terminal and branched chains of chitosan demonstrate strong adsorption energies of − 40.161 and − 71.792 kcal mol^−1^, respectively, aiding in the rapid desolvation of hydrated Zn^2+^ [43]. The calculation results align with the ESP analysis, and both experimental and theoretical findings collectively confirm the capability of chitosan to prevent anions and H_2_O from approaching the zinc anode surface.

Benefiting from the scalability of the scraping strategy, we can successfully and quickly prepare a chitosan/CNTs protective layer on zinc foil (Fig. 1d). The inherent film-forming ability of chitosan enables the formation of a dense, cohesive film after drying, without any additional binders. Therefore, the production cost and time are greatly reduced. Cross-sectional SEM image indicates that the thickness of the protective layer is approximately 16.5 µm (Fig. 1e), and the surface is uniform and flat (Fig. 1f). This uniformity is crucial for achieving consistent zinc deposition and effectively inhibiting the formation of zinc dendrites. Furthermore, the contact angle test showed a significant reduction in the electrolyte’s contact angle at the electrode interface after the introduction of the chitosan/CNTs protective layer (Fig. S6), indicating an obvious improvement at interfacial compatibility between the zinc anode and the electrolyte [44]. This improved compatibility promotes the regulation of Zn^2+^ flux and ensures the uniform deposition of zinc. To evaluate the mechanical properties of the chitosan/CNTs protective layer, we conducted nanoindentation tests (Fig. S7). The results show that it has high mechanical strength, with a high elastic modulus of 12.63 GPa, which is beneficial to the inhibition suppress zinc dendrite growth.

### Reversible Zinc Deposition/Stripping Stability

**Fig. 2:**
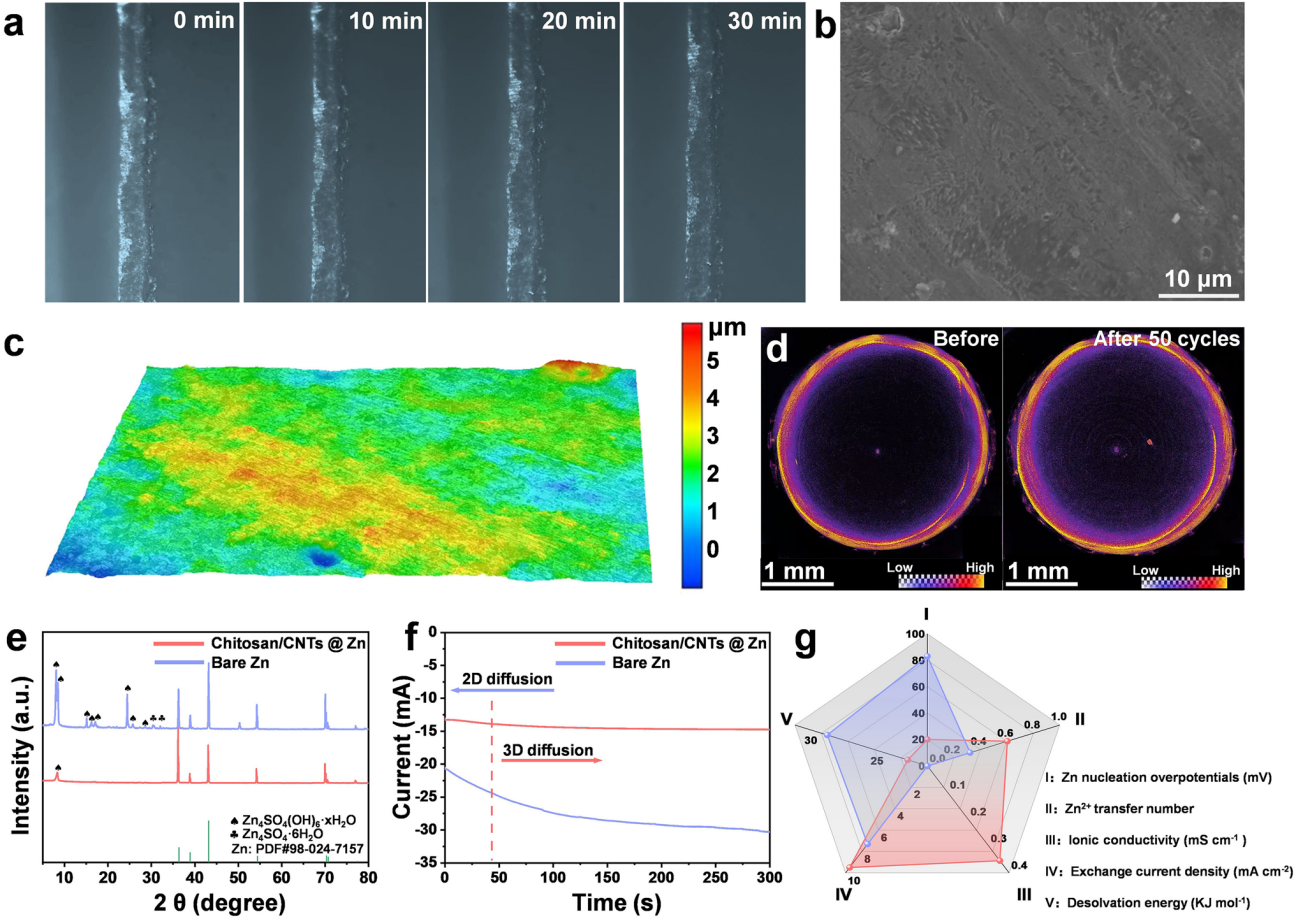
Zn plating/stripping mechanism of chitosan/CNTs @ Zn anode. **a** Optical microscopy images of zinc deposition on chitosan/CNTs @ Zn at the current density of 10 mA cm^−2^. **b** SEM **c** 3D optical image of chitosan/CNTs @ Zn after 20 cycles under 1 mA cm^−2^, 1 mAh cm^−2^. **d** In situ CT before and after circulation of chitosan/CNTs @ Zn. **e** XRD patterns of different anodes after cycling in Zn//Zn symmetrical beaker batteries. **f** Chronoamperograms (CAs) of chitosan/CNTs @ Zn and bare Zn anodes. **g** Radar diagram of electrochemical performance of different anodes

The real-time monitoring of zinc plating/stripping behavior on the electrode surface is critical for understanding this improvement. Using in situ optical microscopy, inhomogeneous deposition was observed on the cross section of the bare Zn anode within 10 min. As plating time increased, this inhomogeneous deposition accumulated and eventually developed into zinc dendrites (Fig. S8). In contrast, when protected by the chitosan/CNTs layer, the zinc surface shows no inhomogeneous deposition throughout the plating/stripping process. (Fig. 2a). This indicates that chitosan/CNTs @ Zn facilitates reversible zinc plating/stripping, enabling the battery to achieve stable cycling performance.

We further examined the symmetric batteries after 20 cycles. As shown in Fig. 2b, the surface of the chitosan/CNTs @ Zn anode remains uniformly flat and smooth, whereas the bare Zn anode exhibited distinct zinc dendrite formation (Fig. S9). These results confirm that the chitosan/CNTs layer mitigates the tip effect, thereby inhibiting zinc dendrite formation. 3D optical microscope analysis further characterized the surface of the zinc anode. The surface of bare Zn was uneven and rough (Fig. S10), with disordered dendrites that increased the Zn/electrolyte contact area, accelerating HER [45]. Conversely, the chitosan/CNTs @ Zn surface keeps almost flat after 20 cycles (Fig. 2c). To avoid potential bias from the limited size of the observations using in situ optical microscopy, we employed in situ CT imaging to gain a comprehensive understanding of the zinc anode overall state. Before cycling, the anode's surface was uniformly flat. However, after 50 cycles, the bare Zn anode developed an uneven surface with numerous “bright spots,” indicative of irreversible zinc dendrite formation during battery cycling (Fig. S11). In contrast, the chitosan/CNTs @ Zn surface maintains its original morphology, with no evidence of zinc dendrite formation (Fig. 2d). This result demonstrates that the protonation of chitosan, combined with the uniform local electric field provided by CNTs [46], promotes uniform zinc deposition and simultaneously inhibits side reactions triggered by H_2_O.

Under identical conditions, real-time monitoring was conducted for 10 h using a beaker-type symmetric battery. As shown in Fig. S12a, b, the bare Zn anode displayed visible inhomogeneous deposition after 10 h of cycling. The chitosan/CNTs @ Zn symmetric battery also exhibits notably lower voltage hysteresis than the bare Zn symmetric battery (Fig. S12c, d). XRD analysis further displays more abundant and intense byproduct peaks on the surface of the bare Zn anode (Fig. 2e), attributed to side reactions induced by H_2_O [39]. SEM analysis confirmed that the chitosan/CNTs @ Zn surface was uniform with no dendritic appearance (Fig. S13a), while the bare Zn surface showed an irregular morphology due to disordered zinc deposition (Fig. S13b). This result illustrates that the chitosan/CNTs layer enables uniform Zn^2+^ deposition and inhibits corrosion and zinc dendrite formation. Fixed voltage testing at − 200 mV further shows almost negligible change in current density for chitosan/CNTs @ Zn surface, indicating a uniform Zn^2+^ deposition facilitated by the chitosan/CNTs protective layer (Fig. 2f). On the bare Zn surface, the current rapidly decreases within 300 s, indicating an accelerated zinc deposition rate [47], often accompanied by an increased active surface area and zinc dendrite formation [48].

Comprehensive electrochemical characterizations during the zinc plating/stripping process further highlights the advantages of the chitosan/CNTs protective layer (Fig. 2g). The nucleation overpotential on the bare Zn surface was 83 mV, which reduced to just 20 mV for chitosan/CNTs @ Zn (Fig. S14), this is because the 3D CNTs matrix, lowers the nucleation barrier for Zn^2+^. Additionally, ion conductivity and Zn^2+^ migration number tests confirmed the superiority of the chitosan/CNTs layer. The Zn^2+^ migration number for chitosan/CNTs @ Zn was 0.609 (Fig. S15), significantly higher than the 0.496 observed for bare Zn. This facilitates rapid Zn^2+^ transport and mitigates zinc dendrite growth induced by concentration polarization [49]. The ionic conductivity of chitosan/CNTs @ Zn was measured at 0.355 mS cm^−1^ (Fig. S16), a remarkable improvement compared to chitosan @ Zn. The exchange current density, closely related to zinc deposition kinetics [50, 51], was also higher for the chitosan/CNTs @ Zn anode (9.51 mA cm^−2^) compared to the bare Zn anode (7.34 mA cm^−2^) (Fig. S17). A higher exchange current density implies lower overpotentials and faster surface charge transfer kinetics at a given total current density. Finally, impedance testing at different temperatures allows us to calculate the desolvation activation energy of the charge transfer process using the Arrhenius equation [52]. The desolvation activation energy for chitosan/CNTs @ Zn anode was 21.49 kJ mol^−1^ (Fig. S18), notably lower than the 27.57 kJ mol^−1^ for the bare Zn anode, indicating that the chitosan/CNTs protective layer expedites Zn^2+^ desolvation and enhances zinc deposition kinetics. To further assess the reaction kinetics, we measured the CV of the symmetric cells of bare Zn and chitosan/CNTs @ Zn (Fig. S19). The higher current densities of chitosan/CNTs @ Zn in both cathodic and anodic peaks than those of bare Zn indicate the accelerated kinetics in both deposition and dissolution of Zn owing to the chitosan/CNTs protective layer. Collectively, these electrochemical results demonstrate that the chitosan/CNTs protective layer provides significant advantages for the zinc anode, contributing to improved cycling stability and overall battery performance.

### Synergistic Suppression of Side Reactions and Zinc Dendrite Formation

**Fig. 3:**
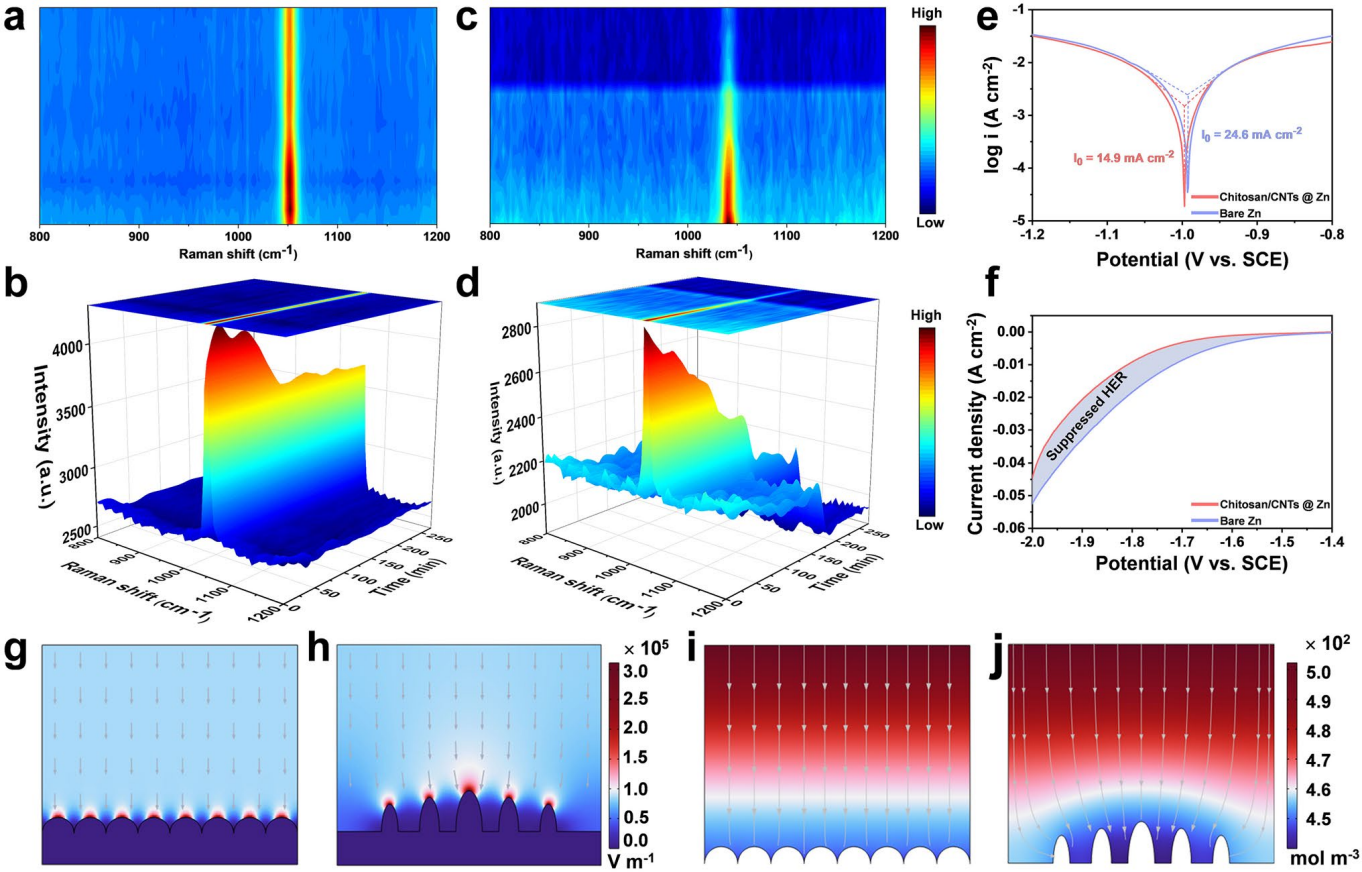
Analysis of inhibition of zinc dendrite and side reaction. In situ Raman spectra of (CF_3_SO_3_)^−^ **a, b** chitosan/CNTs @ Zn anode, **c, d** bare Zn on symmetrical batteries in 2 M Zn(CF_3_SO_3_)_2_. **e** Tafel curves presenting the corrosion status in three-electrode. **f** Linear sweep voltammetry (LSV) profiles. COMSOL electric field distributions simulated **g** chitosan/CNTs @ Zn anode and **h** bare Zn. Zn^2+^ concentration field distributions. **i** Chitosan/CNTs @ Zn anode and **j** bare Zn during the process of Zn deposition

In situ Raman spectroscopy was employed using DEEP-INRS-II to monitor the changes in Zn^2+^ concentration and the stability of H_2_O at the zinc anode interface during the plating process. During discharge, Zn^2+^ migrate to the cathode surface under the influence of the electric field and is reduced to Zn, while the negatively charged (CF_3_SO_3_)^−^ ions migrate in the opposite direction. The Raman signal of (CF_3_SO_3_)^−^near 1050 cm^−1^ can thus serve as an indicator of Zn^2+^ concentration changes [53]. On the chitosan/CNTs @ Zn surface, the stretching vibration peak of (CF_3_SO_3_)^−^ is strengthened during the initial cycle (Fig. 3a, b). This suggests that the desolvation process of Zn^2+^ from the solvation structure composed of Zn^2+^, (CF_3_SO_3_)^−^, and H_2_O alters the chemical environment of (CF_3_SO_3_)^−^. In subsequent cycles, the intensity of (CF_3_SO_3_)^−^ does not show significant fluctuations, indicating that the hydroxyl groups of chitosan can effectively regulate the solvation structure of Zn^2+^ at the electrode/electrolyte interface. This regulation reduces the desolvation energy barrier of Zn^2+^, manages the ionic flux, and promotes the uniform distribution of Zn^2+^ at the electrode/electrolyte interface. Conversely, on the bare Zn surface, the intensity of (CF_3_SO_3_)^−^ decays rapidly (Fig. 3c, d), indicating an uneven Zn^2+^ flux. This leads to increased concentration polarization at the electrode/electrolyte interface. Additionally, the Raman signal at 3500 cm^−1^ corresponds to the stretching vibration peak of the hydroxyl group [54]. On the chitosan/CNTs @ Zn surface, the hydroxyl group signal remained stable with no significant fluctuations (Fig. S20a, b), which indicated that under the protective layer, H_2_O at the zinc anode interface remains stable, preventing side reactions. In contrast, on the bare Zn surface, the intensity of the hydroxyl group signal gradually decreased (Fig. S20c, d), suggesting the occurrence of HER and other side reactions.

Moreover, we further investigated the corrosion inhibition performance of the chitosan/CNTs layer by Tafel test (Fig. 3e). The corrosion current (*I*_corr_) of Zn anodes decreased from 24.6 mA cm^−2^ in bare Zn to 14.9 mA cm^−2^ in chitosan/CNTs @ Zn electrode, indicating enhanced corrosion resistance [55]. The chitosan/CNTs layer effectively inhibits the attack of (CF_3_SO_3_)^−^ ions and H_2_O molecules on the zinc anode, thereby hindering the HER at the electrode interface (Fig. 3f), which is beneficial for the long-term cycling stability of the battery. Furthermore, XRD analysis revealed a more pronounced peak corresponding to the byproduct Zn_x_(OTF)_y_(OH)_2x-y_·nH_2_O on the bare Zn anode surface (Fig. S21) [56]. This suggests more severe side reactions in the absence of the protective layer. To elucidate the role of the chitosan/CNTs protective layer in modulating Zn^2+^ deposition, finite-element simulations were performed to analyze the electric field and concentration field distributions on different anodes. As depicted in Fig. 3g, the interface electric field on the chitosan/CNTs @ Zn surface remains uniform, which helps alleviate the formation of zinc dendrites to some extent [57]. In contrast, the bare Zn surface exhibits uneven protrusions and an erratic electric field distribution (Fig. 3h). This uneven local electric field distribution leads to charge accumulation, favoring Zn^2+^ deposition on these protrusions and creating tip effects. During zinc plating, the chitosan/CNTs @ Zn surface achieves a homogeneous Zn^2+^ flux, as shown in Fig. 3i. The coordination of hydroxyl groups in chitosan with Zn^2+^ promotes uniform Zn^2+^ flow, thereby reducing concentration polarization at the Zn anode. In contrast, the bare Zn surface displays uneven Zn^2+^ concentration (Fig. 3j), which contributes to concentration polarization and the subsequent formation of zinc dendrite [58].

### Zn//Zn Symmetric Cell Cycling Performance

**Fig. 4:**
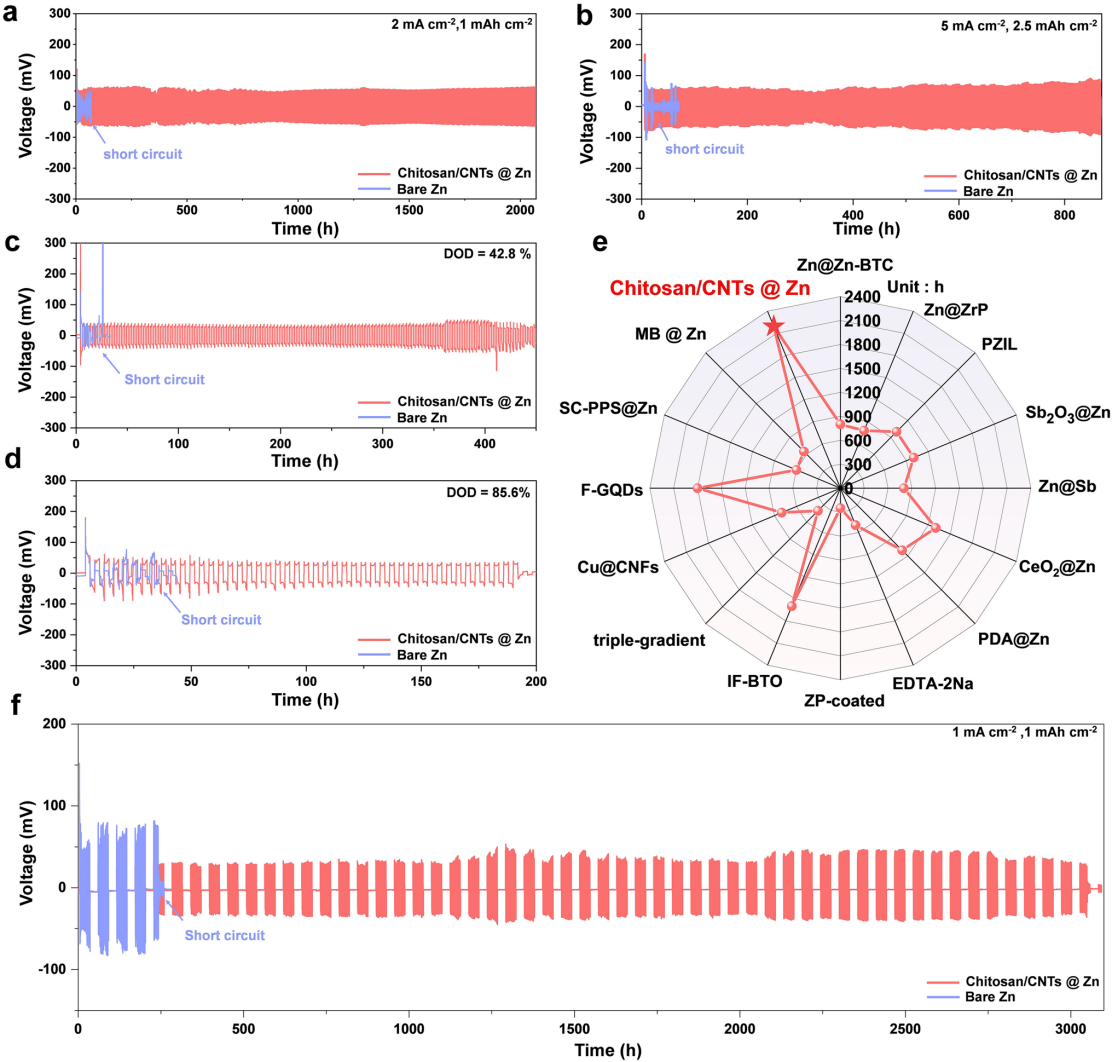
Electrochemical performance for symmetrical batteries. Cycling performance of symmetric batteries with different anodes at **a** 2 mA cm^−2^, 1 mAh cm^−2^ and **b** 5 mA cm^−2^, 2.5 mAh cm^−2^. **c** 42.8% DOD_Zn_ and **d** 85.6% DOD_Zn_. **e** Comparison of our results with the reported data. The details of all the data points are summarized in Table S1. **f** Shelving-recovery performances for different anodes under 1 mA cm^−2^, 1 mAh cm.^−2^

To assess the impact of CNTs content in the chitosan/CNTs protective film on the reversibility of zinc plating/stripping, we conducted cycle stability tests on symmetric batteries at a current density of 1 mA cm^−2^ (Fig. S22). Within a certain range, increasing the CNTs content improved the cycle performance of the battery, owing to the enhanced ionic conductivity of the protective layer with the addition of CNTs. However, excessive CNTs caused agglomeration, and uneven Zn^2+^ deposition. Therefore, the optimal CNTs content was determined to be 100 mg. At a current density of 2 mA cm^−2^, the chitosan/CNTs @ Zn symmetric battery demonstrates remarkable cyclic stability, maintaining performance for over 2000 h with a stable voltage–time curve throughout the cycling process. In contrast, the bare Zn symmetric battery short-circuits after only 20 h (Fig. 4a). Even under more demanding conditions (5 mA cm^−2^), the chitosan/CNTs @ Zn symmetric battery continue to exhibit outstanding cyclic stability with minimal voltage–time curve fluctuations, while the bare Zn symmetric battery short-circuits within a few hours (Fig. 4b). And when at 0.5 mA cm^−2^, 0.25 mAh cm^−2^, it can still run stably for 2200 h (only 50 h without chitosan/CNTs protective layer, Fig. S23). Furthermore, the chitosan/CNTs protective layer remains crucial in ZnSO_4_ electrolytes. As Fig. S24 shown, the chitosan/CNTs @ Zn symmetric battery has much better cycling stability than the bare Zn symmetric battery. Since coin-type batteries use zinc foil directly as the anode, there is typically an excess of zinc. Thus, enhancing zinc utilization is crucial for advancing aqueous zinc-ion batteries toward practical applications. At shallow discharge depths, short-circuiting may occur due to dendrite accumulation, corrosion, and HER as side reactions. While at high discharge depths where zinc utilization is maximized, these side reactions become more prominent [59].

To evaluate the cycling durability of the chitosan/CNTs @ Zn system under deep discharge conditions, we performed cycling stability tests. Initially, with a discharge depth of 42.8% (20 μm Zn foil, 5 mAh cm^−2^), the chitosan/CNTs @ Zn symmetric battery exhibits excellent cycling stability for over 400 h, whereas the bare Zn battery quickly shows voltage fluctuations and eventually short-circuits (Fig. 4c). Even at the discharge depth to 85.6%, the chitosan/CNTs @ Zn symmetric battery continues cycling for nearly 200 h (Fig. 4d), attributed to the synergistic effect of protonated chitosan and CNTs. In stark contrast, the bare Zn symmetric battery quickly encounters irreversible voltage fluctuations. We also conducted rate performance assessments on the symmetric batteries at varying current densities of 0.5, 1, 2, 5, and 10 mA cm^−2^. The chitosan/CNTs @ Zn symmetric battery consistently displayed lower voltage hysteresis compared to the bare Zn symmetric battery (Fig. S25), indicating that the chitosan/CNTs protective layer effectively reduces the energy barrier for zinc plating/stripping reactions. We also evaluated the reversibility of zinc plating/stripping by Zn//Cu asymmetric batteries [60]. At 1 mA cm^−2^ and 1 mAh cm^−2^, the chitosan/CNTs @ Zn//Cu asymmetric battery achieved stable cycling for 500 cycles with 99.4% average coulombic efficiency. In contrast, the bare Zn symmetric battery’s efficiency plummeted after just 200 cycles, indicating irreversible plating/stripping (Fig. S26a). The time–voltage curve for the chitosan/CNTs @ Zn//Cu asymmetric battery showed minimal voltage fluctuations, demonstrating good plating/stripping reversibility (Fig. S26b). Conversely, the bare Zn battery showed significant voltage fluctuations after 70 h of cycling (Fig. S26c), suggesting insufficient active zinc utilization due to side reactions and increased dead zinc formation. Furthermore, the chitosan/CNTs @ Zn//Cu asymmetric battery exhibited lower polarization voltage compared to the bare Zn//Cu asymmetric battery (Fig. S27a, b). These findings demonstrate that the presence of protonated chitosan and CNTs enhances the reversibility of zinc plating/stripping, ensuring high recharging efficiency of the zinc anode. It is worth noting that the Zn//Zn symmetric cell with a chitosan/CNTs composite protective layer exhibits significantly enhanced performance compared to that with a single-layer protective layer. (Fig. 4e). Additionally, we evaluated the role of the protective layer in practical battery operation through shelving-recovery tests. The Zn//Zn battery operated at 1 mA cm^−2^ and 1 mAh cm^−2^, with a 24-h rest after every 15 cycles. The battery with the chitosan/CNTs protective layer can operate stably for over 3000 h, whereas the bare Zn battery lasts less than 245 h (Fig. 4f). These results confirm the outstanding ability of the chitosan/CNTs protective layer to inhibit side reactions, making it suitable for practical applications.

### Electrochemical Performance and Technical–Economic Analysis of Zn//V_2_O_5_ Cells

**Fig. 5:**
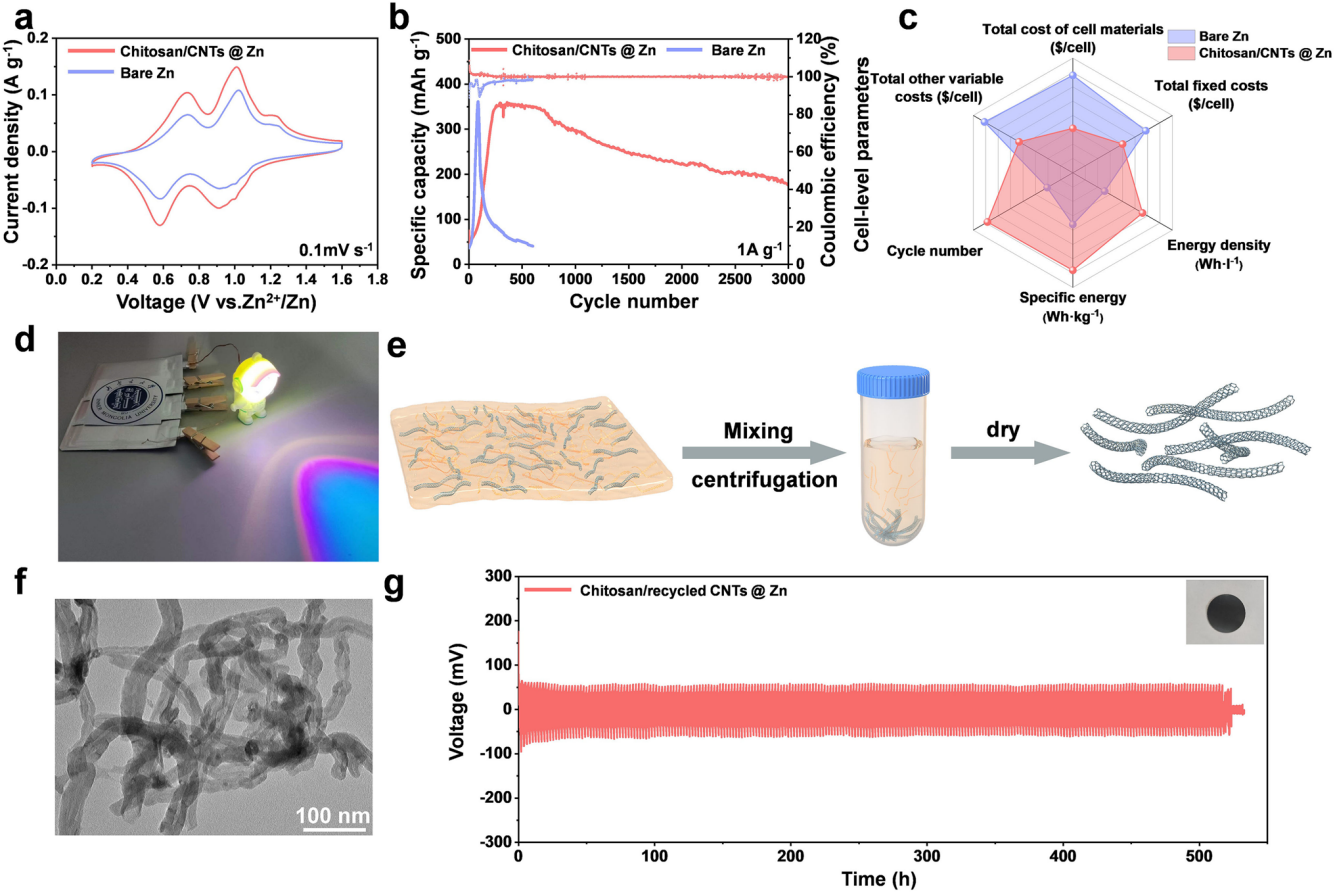
Full batteries performance and CNTs recovery. Electrochemical performance of bare Zn//V_2_O_5_ and chitosan/CNTs @ Zn//V_2_O_5_ batteries. **a** CV curves at 0.1 mV s^−1^. **b** Galvanostatic charge–discharge curves. **c** Improvement of the cell-level metrics (energy density, specific energy, and cost per kWh) when considering a Zn//V_2_O_5_ battery with bare Zn and chitosan/CNTs @ Zn presented in the article. Noted: Total cost of cell materials herein is excluding the cost of the chitosan/CNTs protective layer. **d** Optical photograph of a LED lit by the chitosan/CNTs @ Zn//V_2_O_5_ battery. **e** CNTs recycling schematic diagram. **f** TEM image of extracted CNTs. **g** Cycling performance of chitosan/extracted CNTs @ Zn symmetric batteries under 1 mA cm^−2^, 1 mAh cm^−2^

Subsequently, to investigate the practical applicability of the chitosan/CNTs protective layer, a complete battery was assembled using commercial V_2_O_5_ as the cathode. The electrochemical behavior is characterized by cyclic voltammetry (CV), as shown in Fig. 5a. The CV curves display two pairs of redox peaks, indicative of the Zn^2+^ insertion/extraction process within the V_2_O_5_ structure [23]. Notably, the chitosan/CNTs @ Zn//V_2_O_5_ configuration exhibits higher redox peak currents compared to the bare Zn counterpart, suggesting that the chitosan/CNTs protective layer significantly enhances the reaction kinetics of the battery. The cyclic stability of the battery was tested at a current density of 1 A g^−1^. During the early cycles, there is a noticeable trend of gradually increasing capacity (Fig. 5b), which corresponds to the activation process of V_2_O_5_. The chitosan/CNTs @ Zn//V_2_O_5_ battery not only demonstrates a higher initial capacity but also exhibits superior capacity retention over prolonged cycling. In contrast, the bare Zn//V_2_O_5_ configuration suffers from a rapid capacity decline, attributed to vanadium dissolution and the intrinsic poor conductivity of V_2_O_5_ [61]. Figure S28 presented the rate performance of the full battery across a range of current densities from 0.1 to 5 A g^−1^. Even after cycling at the lowest current density of 0.1 A g^−1^, the chitosan/CNTs @ Zn//V_2_O_5_ battery retained a capacity of 389 mAh g^−1^, significantly higher than the 317 mAh g^−1^ achieved by the bare Zn//V_2_O_5_ battery. This highlights the effectiveness of the chitosan/CNTs protective layer in maintaining high capacity under varying operational conditions. We performed GITT on chitosan/CNTs @ Zn//V_2_O_5_ and bare Zn//V_2_O_5_ batteries and calculated the corresponding diffusion coefficients (Fig. S29). The chitosan/CNTs @ Zn//V_2_O_5_ battery has a higher average diffusion coefficient than the bare Zn//V_2_O_5_ battery, indicating enhanced Zn^2+^ diffusion.

To assess the commercial and industrial viability of battery technology, we undertook a technical and economic analysis focusing on cost and energy density. From an economic cost perspective, the chitosan/CNTs @ Zn//V_2_O_5_ battery demonstrates lower total costs compared to the bare Zn//V_2_O_5_ battery, considering cell material costs, direct labor, variable overhead, and fixed costs (Fig. 5c; detailed data in Tables S2–S4). This cost advantage is further enhanced by the battery’s superior energy density and specific energy, which are critical for high-performance energy storage. Crucially, these assessments assume an optimized production scenario for the protective layer, where its thickness and weight do not affect the mass or volume of the cell—like the solid electrolyte interphase in lithium-ion battery anodes. Additionally, the cost of this layer was assumed to be zero, as estimating production costs at this early research stage is impractical. As such, the difference in costs between the zinc cells with and without the protective layer serves as an upper bound for the layer's cost to ensure the additional treatment does not make the zinc battery more expensive. Moreover, the chitosan/CNTs @ Zn//V_2_O_5_ battery exhibited lower total annual costs, encompassing reduced material, variable, and fixed costs (Fig. S30). These economic benefits are anticipated to substantially lower production expenses, underscoring the promising application value and economic potential of chitosan/CNTs @ Zn batteries. Furthermore, we have successfully assembled soft pack batteries. As shown in Fig. 5d, this pouch battery can power a small robot efficiently, showcasing its applicability in real-world scenarios. Inspired by the impressive performance of the chitosan/CNTs protective layer, we explored its use in other types of metal-ion batteries, such as Al-ion batteries. The cyclic stability of the chitosan/CNTs @ Al symmetric battery was notably superior to that of the bare Al symmetric battery, as shown in Fig. S32. This result underscores the broad applicability and potential of the chitosan/CNTs protective layer for stabilizing various metal-ion battery anodes.

Producing and preparing CNTs is not just expensive because of the raw materials but also requires a lot of energy. This makes recycling CNTs important, not only for saving money, but also for protecting the environment and conserving resources. Therefore, it is very important to develop a method for recovering CNTs from the chitosan/CNTs protective layer in used batteries (i.e., post-cycle). Chitosan has good solubility in glacial acetic acid, but the CNTs in protective layer are not soluble in glacial acetic acid. Hence, we can successfully extract CNTs from the post-cycle chitosan/CNTs protective layer through a process involving crushing, redissolution in glacial acetic acid, stirring, and centrifugation (Fig. 5e). The extracted CNTs (Fig. 5f) are then compared to the original CNTs (Fig. S33) using TEM, and the two are found to be morphologically identical, confirming the successful extraction of CNTs. The recycled CNTs and chitosan were used to fabricate a new protective layer, which was then assembled into a battery. Remarkably, the battery demonstrated stable cycling for over 500 h (Fig. 5g, inset: chitosan/extracted CNTs @ Zn), illustrating the sustainability and reusability of the chitosan/CNTs protective layer. This combination of high cycle stability, adaptability, and sustainable utilization suggests that the chitosan/CNTs protective layer has considerable potential for a wide range of applications in battery technology.

## Conclusions

In this work, we developed a low-cost, multifunctional chitosan/CNTs composite protective layer that addresses critical challenges of zinc anodes in aqueous batteries. By leveraging chitosan’s exceptional film-forming ability and strong adhesion, we seamlessly integrated it with conductive CNTs to create a synergistic interface. The abundant polar functional groups in chitosan facilitate efficient Zn^2+^ transport, while the uniformly distributed CNTs network homogenizes the electric field and reduces local current density, enabling dendrite-free Zn deposition. This dual-action mechanism endows the chitosan/CNTs @ Zn symmetric cell with exceptional cycling stability, achieving over 3000 h of stable operation at 1 mA cm^−2^—a performance that surpasses bare Zn anodes and most reported protective layers.

Notably, the solubility of chitosan in mild acidic conditions allows for convenient recovery and reuse of CNTs, enhancing the sustainability and circularity of zinc-ion batteries. Techno-economic analyses further validate the cost-effectiveness and scalability of this approach, with chitosan’s low cost (~ $10/kg) and the simple scraping technique offering a practical pathway for industrial adoption. These findings highlight the chitosan/CNTs composite as a versatile and eco-friendly solution for stabilizing metal anodes, positioning it as a promising candidate not only for zinc-based batteries, but also for a broad range of next-generation energy storage systems seeking to balance performance, sustainability, and economic viability.

## Supplementary Information

Below is the link to the electronic supplementary material.

Supplementary file1 (DOCX 3519 kb)
