# Supplementary material for: Scalable and Sustainable Chitosan/Carbon Nanotubes Composite Protective Layer for Dendrite-Free and Long-Cycling Aqueous Zinc-Metal Batteries
Source: Nanomicro Lett. 2025 Jul 8;17:326. doi: 10.1007/s40820-025-01837-7 (PMC12238440; doi:10.1007/s40820-025-01837-7)
Supplement: Supplementary file 1 — Supplementary file1 (DOCX 3519 kb) [file 40820_2025_1837_MOESM1_ESM.docx]

Supporting Information for

**Scalable and Sustainable Chitosan/Carbon Nanotubes Composite Protective Layer for Dendrite-Free and Long-Cycling Aqueous Zinc-Metal Batteries**

Jinchang Wang^1^, Alessandro Innocenti^2^, Hang Wei^1, 3^*, Yuanyuan Zhang^1^*, Jingsong Peng^1^, Yuanting Qiao^4^*, Weifeng Huang^3^, Jian Liu^1, 5^*

^1^College of Chemistry and Chemical Engineering, Inner Mongolia Key Laboratory of Rare Earth Catalysis, College of Energy Material and Chemistry, Institute for Green Chemistry and Environmental Science, Inner Mongolia University, Hohhot, Inner Mongolia 010021, P. R. China

^2^Zentrum für Sonnenenergie- und Wasserstoff-Forschung Baden-Württemberg, 89081, Ulm, Germany

^3^China-Italy Joint Laboratory of In-Situ/Operando Instrumentation Beijing Science Star Technology Co. Ltd.

^4^Department of Chemical Engineering, Faculty of Engineering, Swansea University, Swansea SA1 8EN, United Kingdom

^5^DICP-Surrey Joint Centre for Future Materials, Department of Chemical and Process Engineering and Advanced Technology Institute, University of Surrey, Guildford, Surrey, GU2 7XH UK

*Corresponding authors. E-mail: [Yuanting.qiao@swansea.ac.uk](mailto:Yuanting.qiao@swansea.ac.uk) (Yuanting Qiao); [weihang@imu.edu.cn](mailto:weihang@imu.edu.cn) (Hang Wei); [zyy@imu.edu.cn](mailto:zyy@imu.edu.cn) (Yuanyuan Zhang); [jian.liu@surrey.ac.uk](mailto:jian.liu@surrey.ac.uk) (Jian Liu)

**S1 Supplementary Text**

**S1.1 Chemicals**

Chitosan (Aladdin, degree of deacetylation ≥ 95 %), Zn(CF_3_SO_3_)_2_ (Aladdin, ≥ 98 %), acetic acid (Tianjin Xinbote Chemical Co., Ltd, ≥ 95 %), Multi-CNTs (Shenzhen Nanoport Co., Ltd, > 97 %), V_2_O_5_ (Aladdin,> 99.99 %), Super P (TIMICAL), PVDF (Solef 5130), AlCl_3_ (Aladdin,> 99 %), 1-ethyl-3-methylimidazolium chloride (AIYAN, 97 %), NMP (Aladdin, ≥ 99.5 %) were used as received without any purification. Zn foil (100 μm, 20 μm), Cu foil (10 μm), Al foil (100 μm), Carbon paper (TGP-H-060, 0.19 mm).

**S1.2 Electrochemical testing**

All battery tests were conducted using CR2032 model coin cells. Galvanostatic charge/discharge cycling, rate performance, nucleation overpotential, and coulombic efficiency were evaluated using a Neware electrochemical system. Cyclic voltammetry (CV) was performed at a scan rate of 0.1 mV s⁻^1^, and electrochemical impedance spectroscopy (EIS) was measured in the frequency range of 0.01-100 kHz (The potential control mode was employed to test the battery at its open-circuit potential). Chronoamperograms and linear polarization curves were obtained using an electrochemical workstation (CHI760E). All electrochemical tests except for temperature-variable impedance spectroscopy were conducted at room temperature.

The Zn²⁺ transference number was determined using the steady-state current method, with EIS measurements taken before and after the chronoamperometry. The value of Zn^2+^ transference number was calculated by the equation (S1) as follow:

$t_{{Zn}^{2+}}=\frac{I_{s}\left( \Delta V-I_{0}R_{0} \right)}{I_{0}\left( \Delta V-I_{s}R_{s} \right)}$ (S1)

where *ΔV* is the applied voltage polarization (10 mV), *R_0_* and *R_s_* represent the impedance before and after chronoamperometry test while *I_0_* and *I_s_* represent initial and stable current during polarization.

The ionic conductivity of the protective film was measured with EIS using a stainless steel electrode/protective film/stainless steel electrode system. The conductivity is calculated by the equation (S2) as follow:

$\sigma=\frac{L}{R_{b} S}$ (S2)

where *R_b_* represents the bulk resistance according to the EIS measurements, *L* represents the thickness of the protective film, and *S* is the contact area.

The activation energy *E_a_* was calculated by Arrhenius equation (S3):

${1/R}_{ct}=A e^{\left( \frac{-E_{a}}{RT} \right)}$ (S3)

where *A* is the frequency factor, *R* is the gas constant, *R_ct_* is the interface resistance, and *T* is the absolute temperature (20-70°C).

Calculation of the depth of discharge (DOD) was done with the equation (S4) as follows:

$$DOD=\frac{3.6 X M}{\rho N_{a} n e l} (S4)$$

where 𝝆 is the zinc density, *N_a_* is the Avogadro’s constant (*N_a_* = 6.02×10^23^), *n* is the number of electrons transferred for the Zn^0^ to Zn^2+^ conversion (*n* = 2), *e* is the electron electric charge (1.6×10^-19^ C) and *l* is the thickness of the zinc foil (*l* = 20 µm). *X* represent the areal capacity in each half cycle during Zn stripping/plating and *M* is the molecular mass of Zn (*M* = 65.38 g mol^-1^). According to this equation, the *DOD* = 42.8 % when the areal capacity is 5 mAh cm^-2^, while for an areal capacity of 10 mAh cm^-2^ the *DOD* = 85.6 %.

Equivalent circuit models for impedance data:


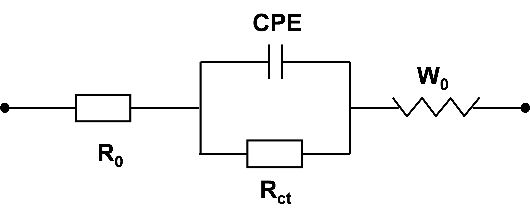


GITT test method:

The surface diffusion coefficient of Zn^2+^ is measured by Zn//V_2_O_5_ full batteries. The cells are charged and discharged intermittently with a current density of 0.2 A g^-1^, the charge-discharge time is 300 s, and the relaxation time is 30 min. The diffusion coefficient *D_Zn_^2+^* is obtained by the following formula:

*D_Zn_^2+^* = $\frac{4}{\Pi\tau} \left（ \frac{m_{B}V_{M}}{M_{B}S} \right）^{2}$( $\frac{\Delta E_{S}}{\Delta E_{\tau}}$) (S5)

where 𝜏 is the duration of the current pulse; 𝑚𝐵, 𝑉_𝑀_, 𝑀_𝐵_ and 𝑆 are the mass of the active material, the molar volume, the molar weight and the electrochemical active area respectively. ∆E*_S_* is the total voltage change caused by pulse and ∆E_𝜏_ is the voltage change of constant current charge/discharge.

**S1.3 Theoretical calculations**

**Density Functional Theory (DFT) calculation.** DFT was employed to calculate the energy levels of H₂O and chitosan molecules. The structures of H₂O, (CF₃SO₃)⁻, and chitosan were optimized using Gaussian 09 with the D1 method at the B3LYP/6-31G* level of theory. The structure of Zn^2^⁺ was optimized using Gaussian 09 with the D1 method at the B3LYP/SDD level of theory. The restrained electrostatic potential (RESP) atom charges and electrostatic potential (ESP) were calculated using Multiwfn 3.8 software [S1].

**Finite-element simulations.** The electrochemical reaction process of Zn^2+^ on the anodic deposition interface of zinc ion battery was simulated by finite element analysis method using the software COMSOL Multiphysics^®^. In these simplified simulations, the migration of Zn^2+^ driven by electric field and diffusion flow in liquid phase (electrolyte) and solid phase is considered. The length of the electrodes is 8 μm, and the distance between them is 5.5 μm. The bulge on the surface of the bare Zn anode is composed of five ellipses (radius is 0.1 μm), and the distance between the two semicircles is 1 μm. The cathode potential is set to 0.5 V, and the anode potential is set to 0 V. The concentration of Zn^2+^ was calculated by the Nernst-Planck equation. In this model, the voltage difference of the electrode was set to 0.5 V. The initial Zn^2+^ concentration was set to 2 M. The diffusion coefficient of Zn^2+^ in the electrolyte was set to 2×10^-10^m^2^ s^-1^. The average current density through the cell is set to 225 A m^-2^.

**Simulations of battery cost and energy density.** The evaluation of energy density and costs was conducted using the open-source BatPaC 5.0 software. A comprehensive overview of the BatPaC model is available in the associated report published by Argonne National Laboratory [S2]. This model is tailored for simulating battery packs with specific energy and power requirements, taking into consideration the costs of various elements, including active materials, conductive carbon, binders, separators, electrolytes, current collectors, casings, pack current collectors, cooling systems, labor, and overhead expenses. It also includes the capital costs associated with setting up the production site.

The battery pack simulated is intended for residential energy storage, with a power rating of 1 kW and an energy rating of 11.5 kWh. Each module contains 72 cells, arranged in a series of two modules, resulting in a total of 144 cells per battery pack. The production volume is targeted at 25,000 packs annually. The cathode active material cost was assumed in accordance with the current bulk prices of vanadium pentoxide [S3], while the rest of the costs and parameters was based on the work of Innocenti et al [S4]. The batteries were simulated using the voltage vs. specific capacity discharge curve of Fig. S31 (curves @ 0.1 A·g^-1^). The costs associated with the dry room and the production of the negative electrode were excluded for the rechargeable zinc battery. The aqueous electrolyte removes the need for a dry environment during materials and battery processing. Furthermore, the negative electrode, made from zinc metal foil, does not require coating or drying procedures. All primary parameters for the simulations are provided in Table S2, with the key results shown in Table S3.

The simulations are performed assuming a hypothetical optimized rechargeable zinc battery system, as the metrics reported in the main texts, such as the amount of active material in the cathode, zinc electrode utilization, and thickness of the components, are not suitable for a commercial system. Specifically:

•The chosen electrode composition, electrode porosity, separator thickness are representative of typical commercial lithium-ion batteries.

• The electrolyte is assumed to be a 2 M ZnSO_4_ solution in water [S4]. In fact, the zinc triflate used as salt in the electrolyte, being a specialty chemical, is too expensive to be practically considered as electrolyte salt for these calculations [S4].

• No additional cost was assumed for the protective layer. This allows us to determine the maximum cost at which this extra treatment could be added without negating the cost benefits per kWh. Specifically, the cell cost decreases from $162 to $151 per kWh, meaning the protective layer should not exceed $11 per kWh to remain cost-effective. However, this calculation does not account for the substantial improvement in battery cycle life provided by this treatment.

•We had to assume a maximum power output of 1 kW instead of 7 kW, as used in other studies simulating similar small battery packs. The relatively low average voltage of these zinc batteries (about 0.77 V) necessitates a much higher nominal cell current to deliver sufficient power. Stainless steel current collectors, commonly used in rechargeable aqueous zinc batteries, have a resistivity that is an order of magnitude higher than that of copper or aluminum used in lithium-ion batteries. This increased resistivity, coupled with the high current on the thin 20 µm sheets assumed in our calculations, would make it impractical to assume a 7 kW maximum power output. In that scenario, current collectors around 500 µm thick would be required to supply such power, but this would drastically reduce the cell’s energy density and specific energy.

S2 Supplementary Figures and Tables


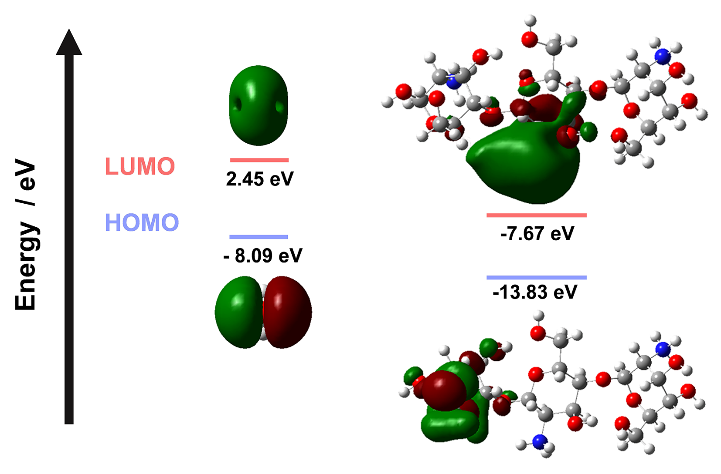


**Fig. S1** Molecular orbital energies of H_2_O and chitosan molecules calculated by DFT

**
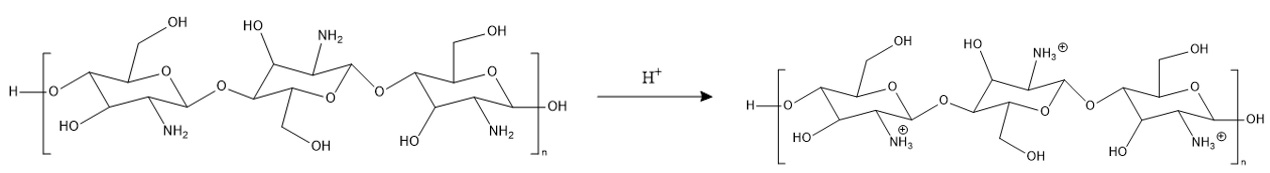
**

**Fig. S2** Chitosan amino protonation reaction formula

**
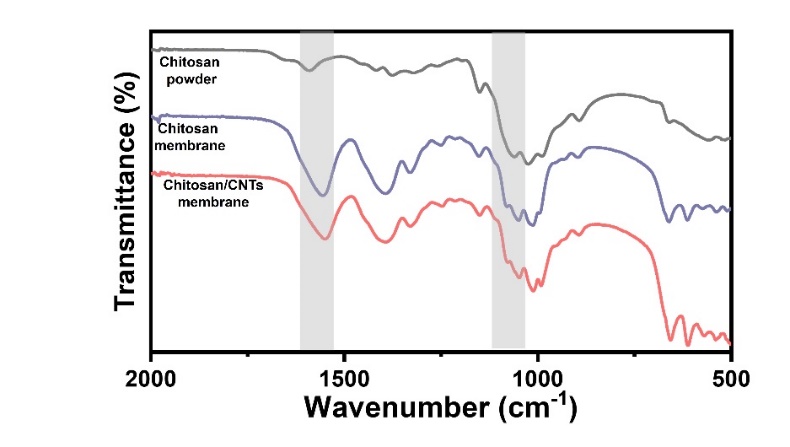
**

**Fig. S3** FT-IR spectrum of chitosan powder, chitosan and chitosan/CNTs protective layer


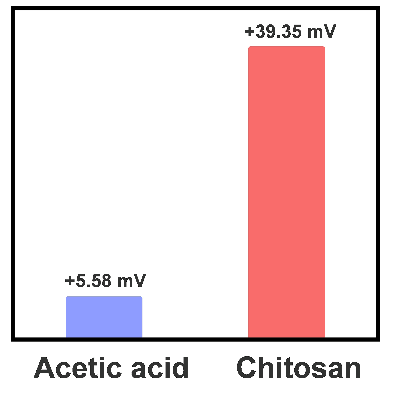


**Fig. S4** Zeta potentials of acetic acid and chitosan dissolved in acetic acid


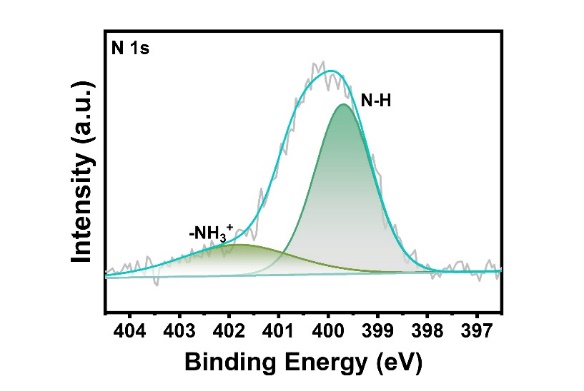


**Fig. S5** XPS spectra of N 1s for chitosan/CNTs protective layer


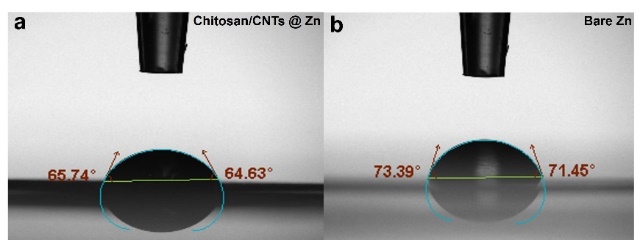


**Fig. S6** Contact angles of 2 M Zn(CF_3_SO_3_)_2_ electrolyte on bare Zn and chitosan/CNTs @ Zn


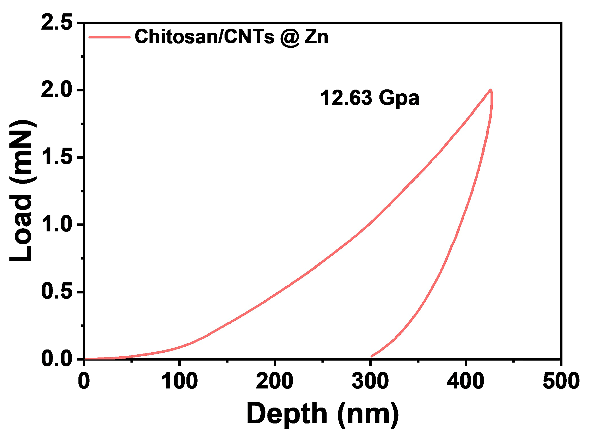


**Fig. S7** Mechanical strength of the chitosan/CNTs protective layer


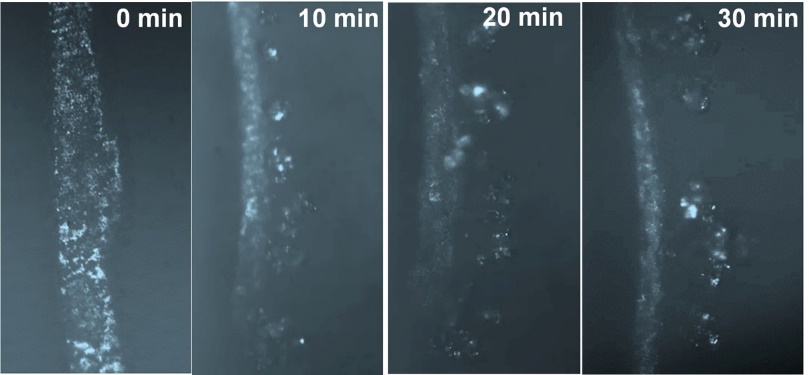


**Fig. S8** Optical microscopy images of zinc deposition on bare Zn at 10 mA cm^-2^.


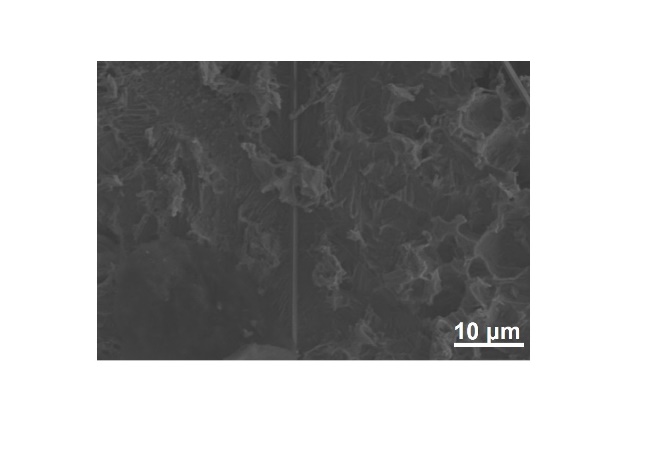


**Fig. S9** SEM of bare Zn after 20 cycles under 1 mA cm^-2^,1 mAh cm^-2^


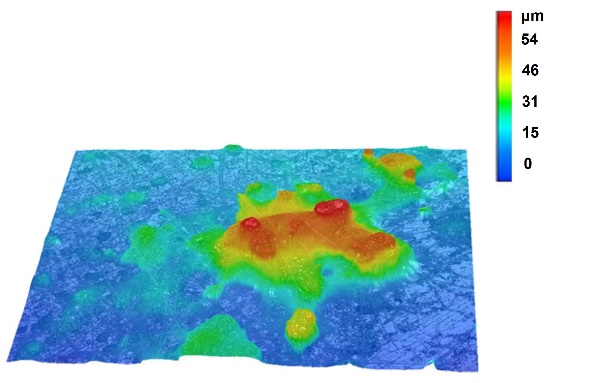


**Fig. S10** 3D optical image of bare Zn after 20 cycles under 1 mA cm^-2^, 1 mAh cm^-2^


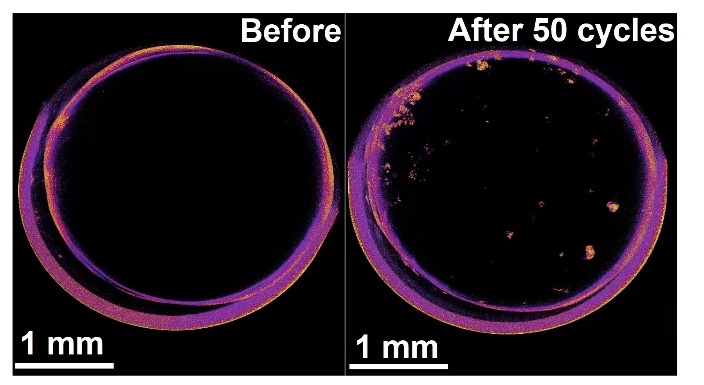


**Fig. S11** In situ CT before and after cycling of bare Zn


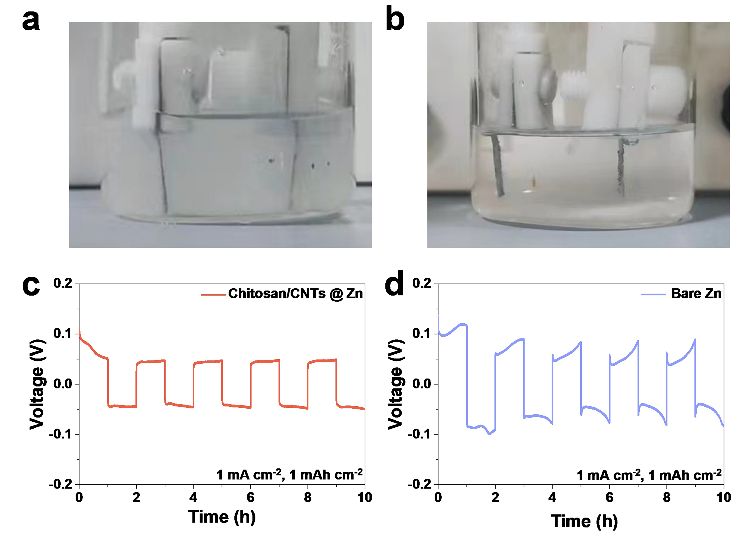


**Fig. S12** Zn//Zn symmetric beaker batteries digital camera images (**a**) chitosan/CNTs @ Zn and (**b**) bare Zn anode. Cycle performance of Zn // Zn symmetric beaker batteries (**c**) chitosan/CNTs @ Zn and (**d**) bare Zn anode


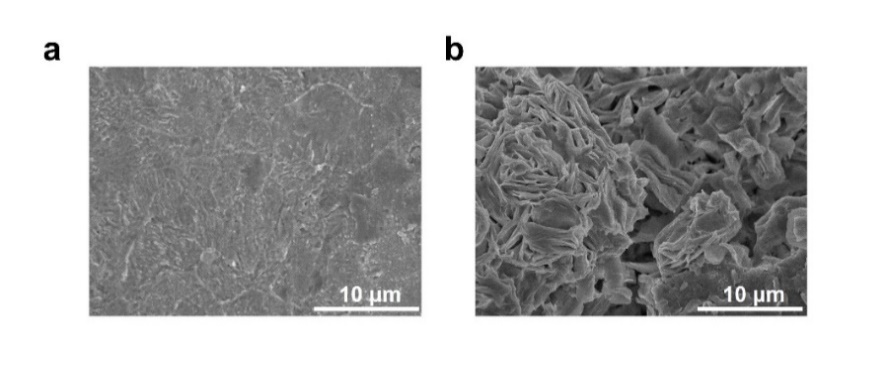


**Fig. S13** SEM of different anodes after cycling in Zn//Zn symmetrical beaker batteries (**a**) chitosan/CNTs @ Zn (**b**) bare Zn


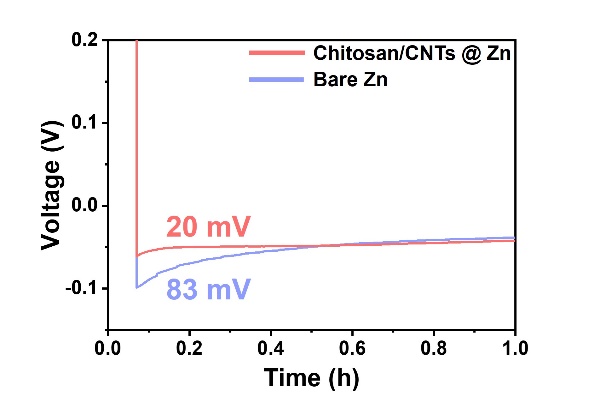


**Fig. S14** Nucleation overpotential for Zn//Cu batteries with chitosan/CNTs @ Zn and bare Zn anodes at 1 mA cm^-2^, 1 mAh cm^-2^


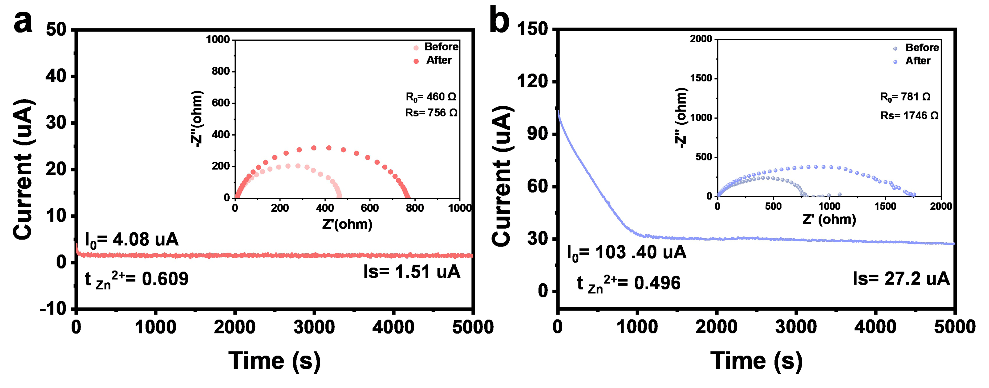


**Fig. S15** Current–time curve of (**a**) chitosan/CNTs @ Zn and (**b**) bare Zn symmetric batteries in 2 M Zn(CF_3_SO_3_)_2_ at 10 mV. Inset: the electrochemical impedance spectroscopy (EIS) spectra of the chitosan/CNTs @ Zn and bare Zn symmetric batteries before and after the CA test


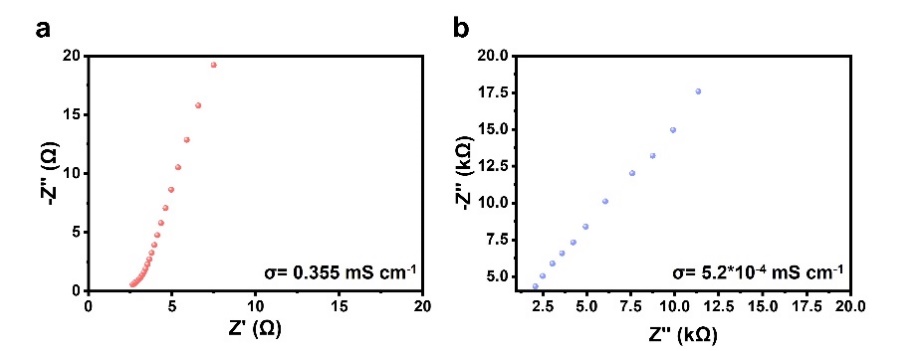


**Fig. S16** Electrochemical impedance spectra of (**a**) chitosan/CNTs and (**b**) chitosan protective layer


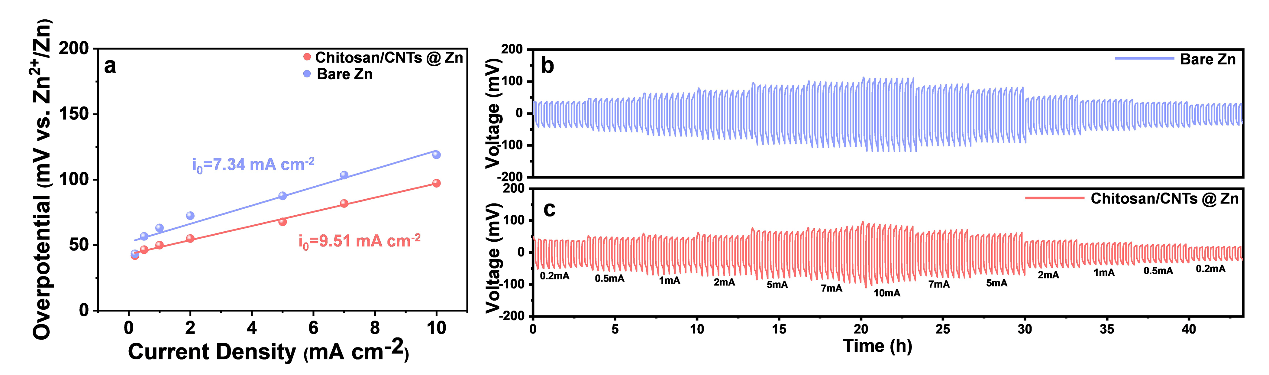


**Fig. S17** (**a**) Exchange current density comparison of chitosan/CNTs @ Zn and bare Zn anodes. The (**b**) bare Zn (**c**) chitosan/CNTs @ Zn symmetrical battery was cycled for 10 cycles at 0.2 mA cm^-2^ to 10 mA cm^-2^


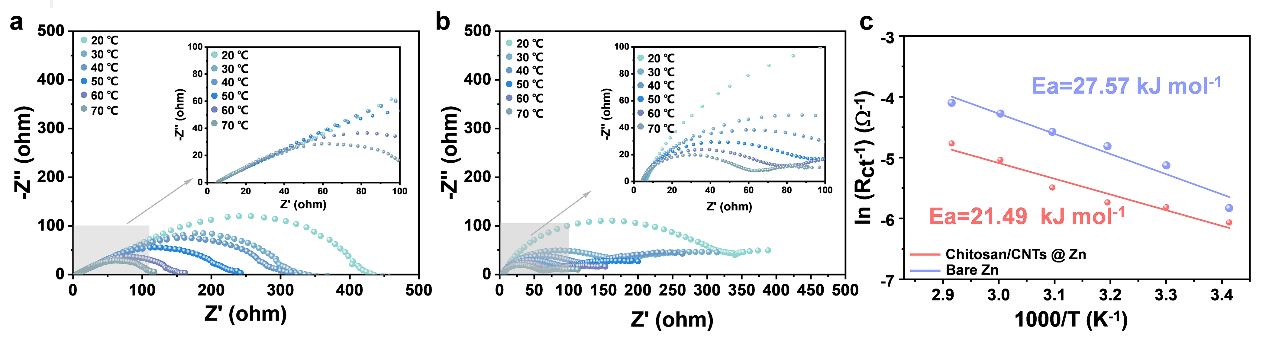


**Fig. S18** EIS spectra at different temperatures of Zn//Zn symmetric batteries with (**a**) chitosan/CNTs @ Zn and (**b**) bare Zn anode. (**c**) The calculated activation energy of different anodes


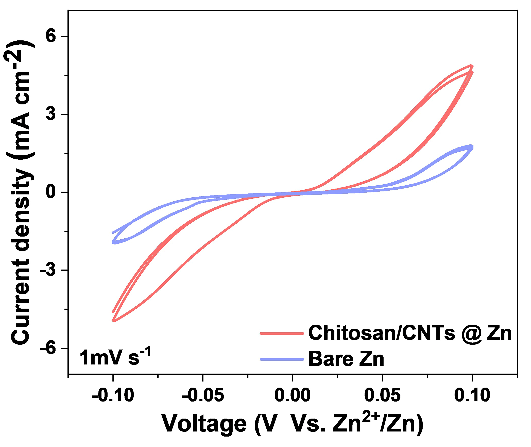


**Fig. S19** CV curves of symmetric bare Zn and chitosan/CNTs @ Zn batteries at a scan rate of 1 mV s^−1^


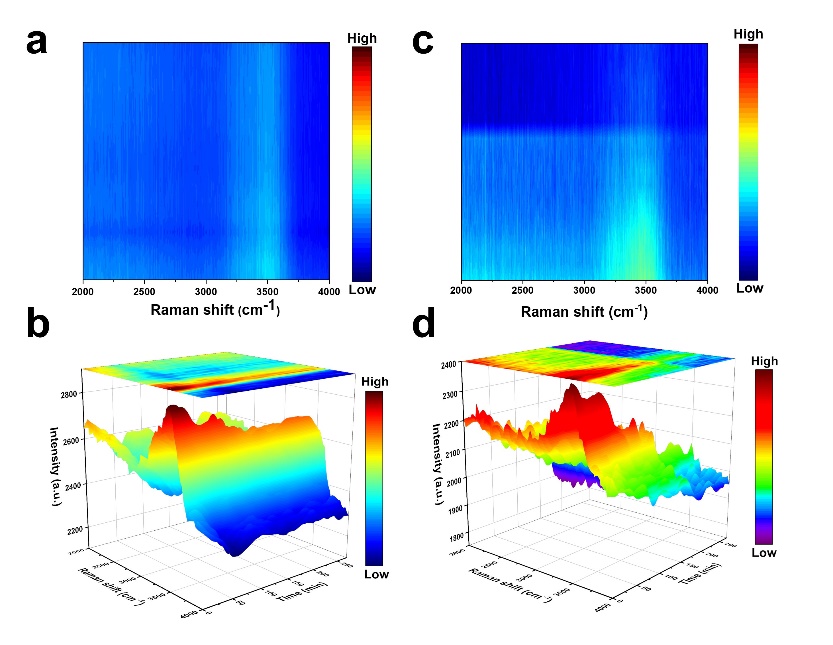


**Fig. S20** Two-dimensional and three-dimensional in-situ Raman spectra of -OH (a,b) chitosan/CNTs @ Zn (c,d) bare Zn on symmetrical batteries in 2 M Zn(CF_3_SO_3_)_2_


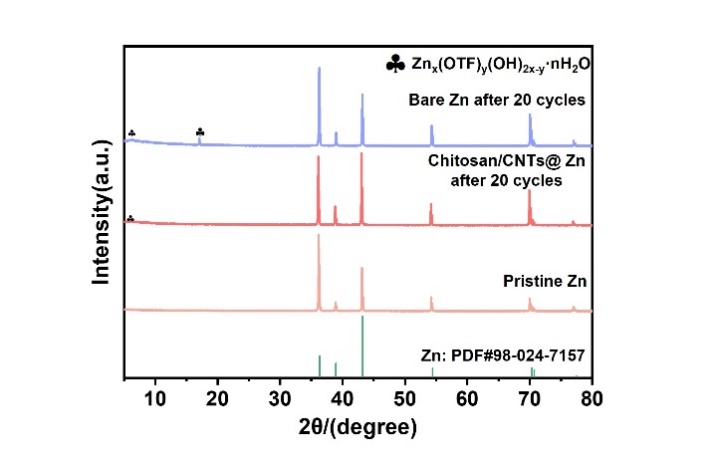


**Fig. S21** XRD patterns of chitosan/CNTs @ Zn and bare Zn anodes after 20 cycles under 1 mA cm^-2^, 1 mAh cm^-2^


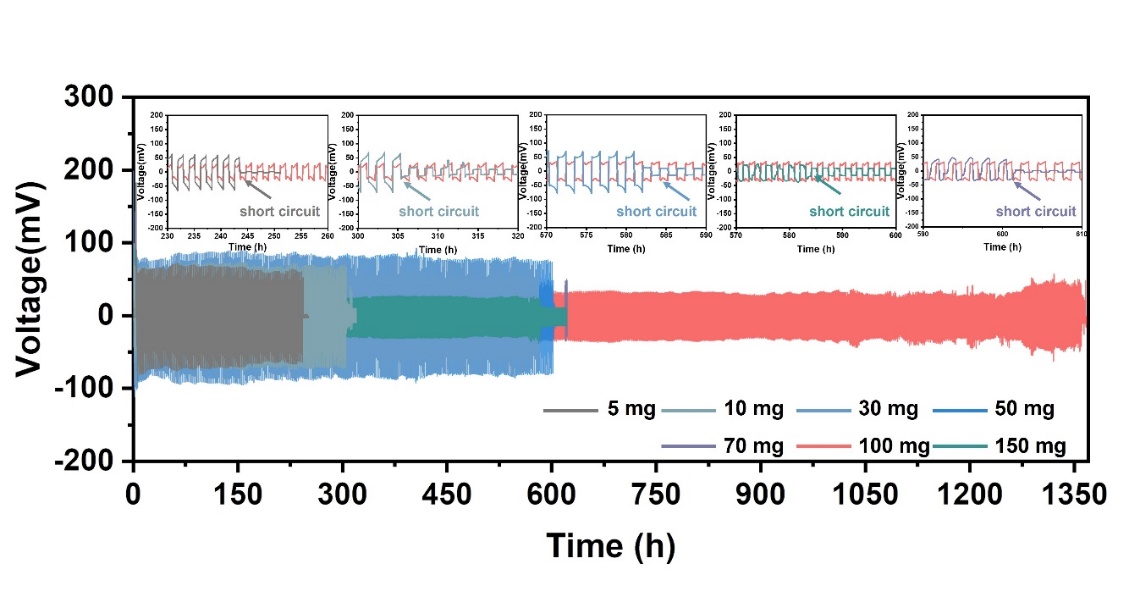


**Fig. S22** Cycling performance of chitosan/CNTs @ Zn symmetrical battery with different CNTs at 1 mA cm^-2^, 1 mAh cm^-2^


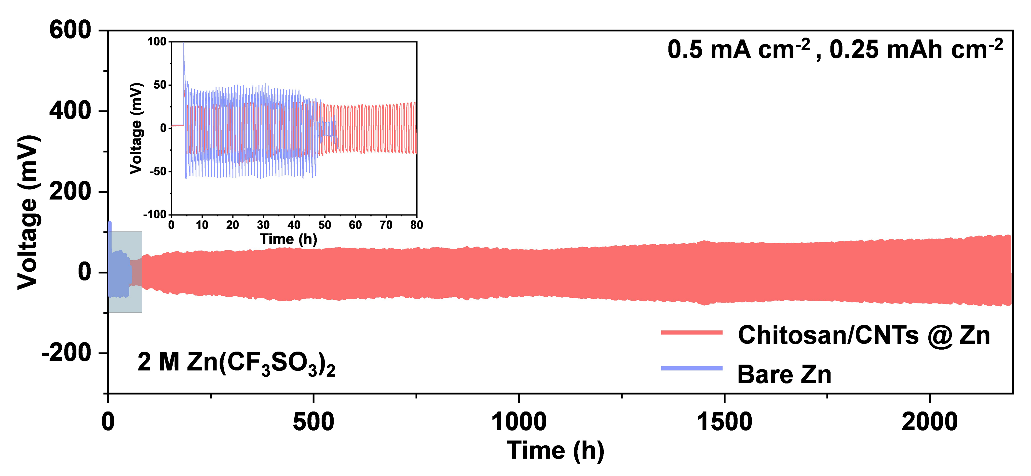


**Fig. S23** Cycling performance of symmetric batteries with different anodes at 0.5 mA cm^-2^, 0.25 mAh cm^-2^


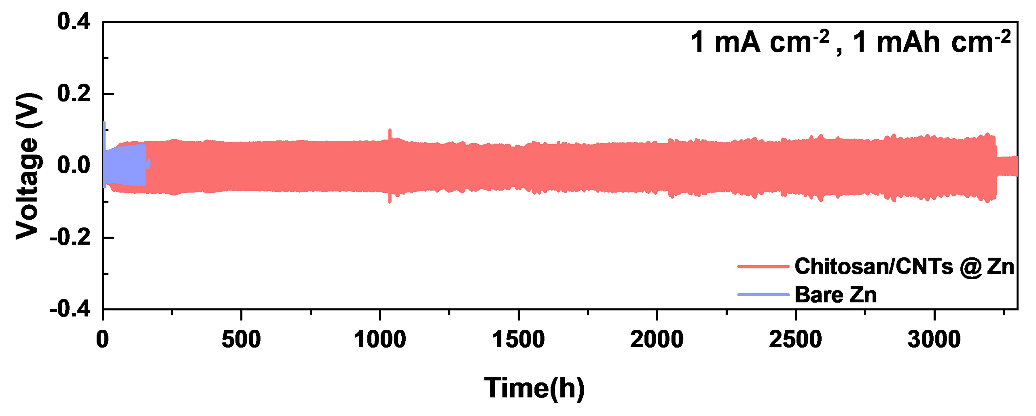


**Fig. S24** Cycling performance of symmetric batteries with different anodes in 2 M ZnSO_4_ at 1 mA cm^-2^, 1 mAh cm^-2^


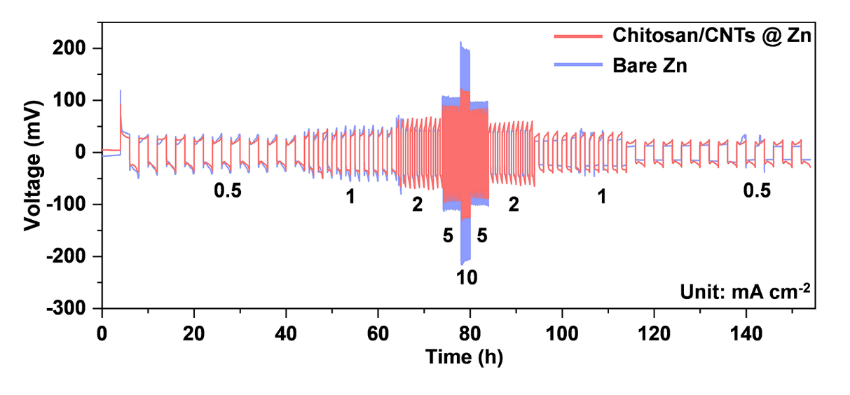


**Fig. S25** Rate performance of symmetric batteries with different anodes and the corresponding voltage hysteresis was obtained at different current densities from 0.5 to 10 mA cm^-2^


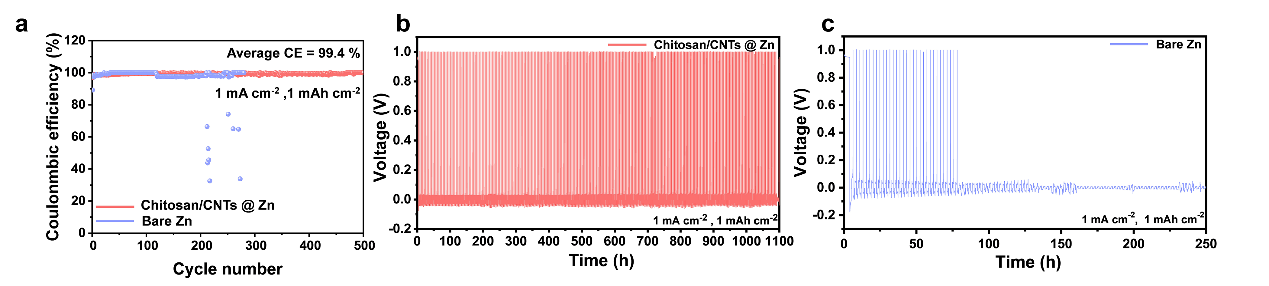


**Fig. S26** (**a**) Coulombic efficiencies of Zn//Cu and chitosan/CNTs @ Zn//Cu half batteries during cycles at 1 mA cm^-2^. Corresponding voltage profiles of the asymmetric (**b**) chitosan/CNTs @ Zn//Cu and (**c**) bare Zn//Cu batteries


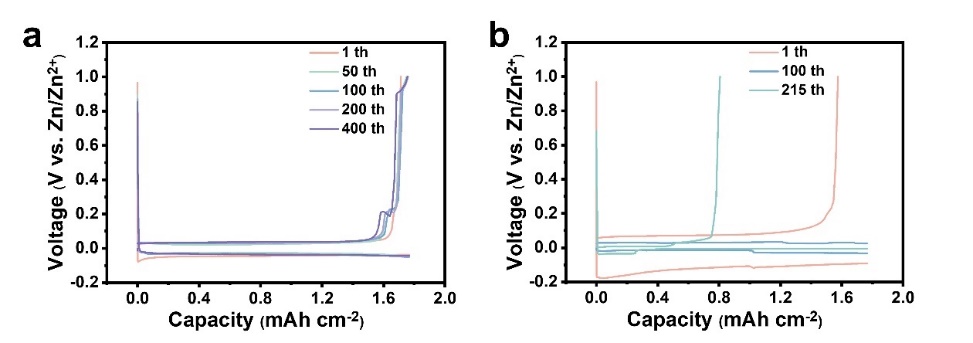


**Fig. S27** GCD profiles of the asymmetric (**a**) chitosan/CNTs @ Zn//Cu and (**b**) bare Zn @ Cu batteries at 1 mA cm^-2^, 1 mAh cm^-2^


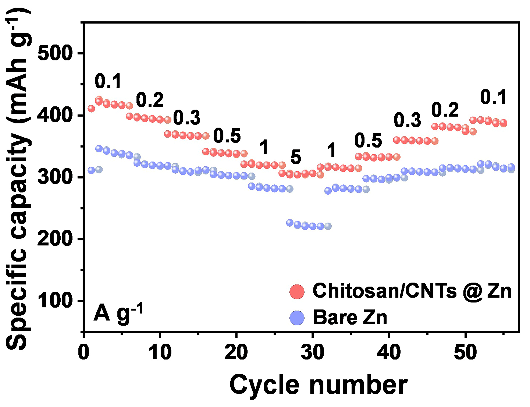


**Fig. S28** Rate performance at different rates of bare Zn// V_2_O_5_ and chitosan/CNTs @ Zn//V_2_O_5_ batteries


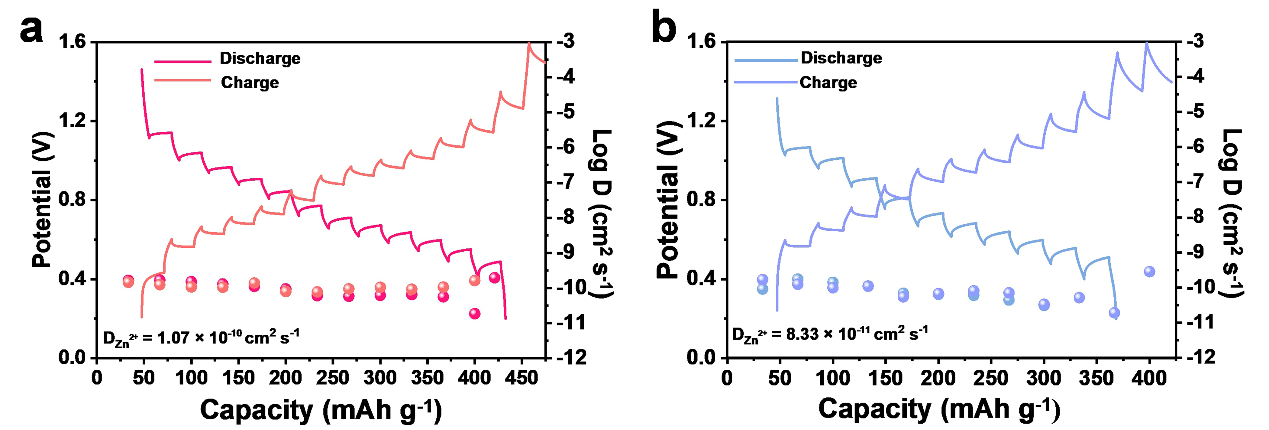


**Fig. S29** GITT curves and corresponding diffusion coefficient of (a) chitosan / CNTs @ Zn//V_2_O_5_ and (b) bare Zn//V_2_O_5_ batteries


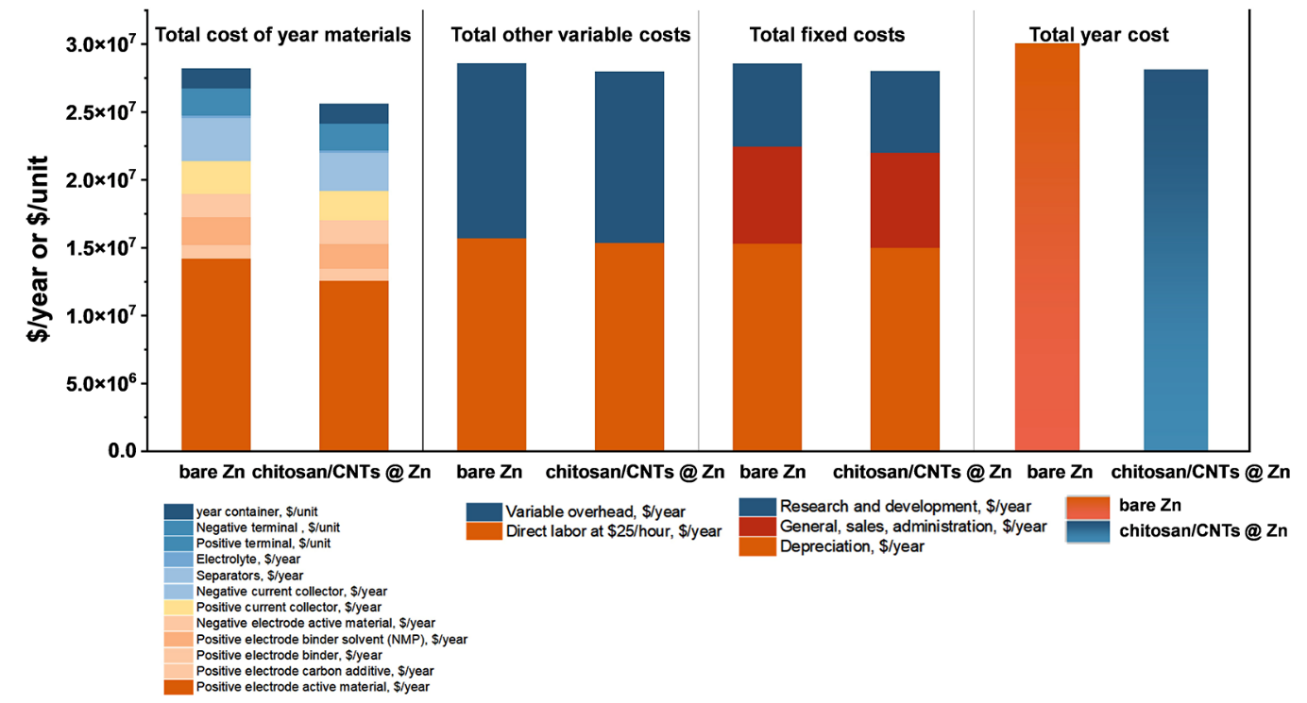


**Fig. S30** Bare Zn// V_2_O_5_ and chitosan/CNTs @ Zn//V_2_O_5_ batteries annual cost assessment of production materials. Noted: Total cost of cell materials herein is excluding the cost of the chitosan/CNTs layer. Noted: Total cost of cell materials herein is excluding the cost of the chitosan/CNTs protective layer


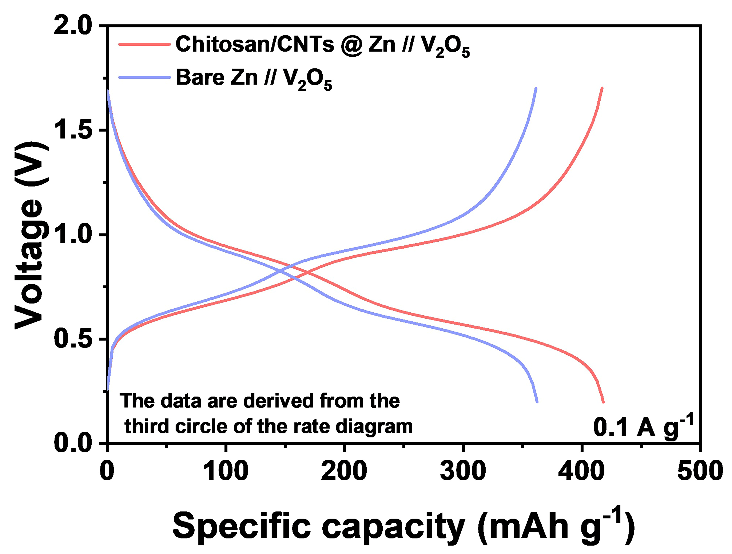


**Fig. S31** The 3^st^ GCD of bare Zn// V_2_O_5_ and chitosan/CNTs @ Zn//V_2_O_5_ batteries


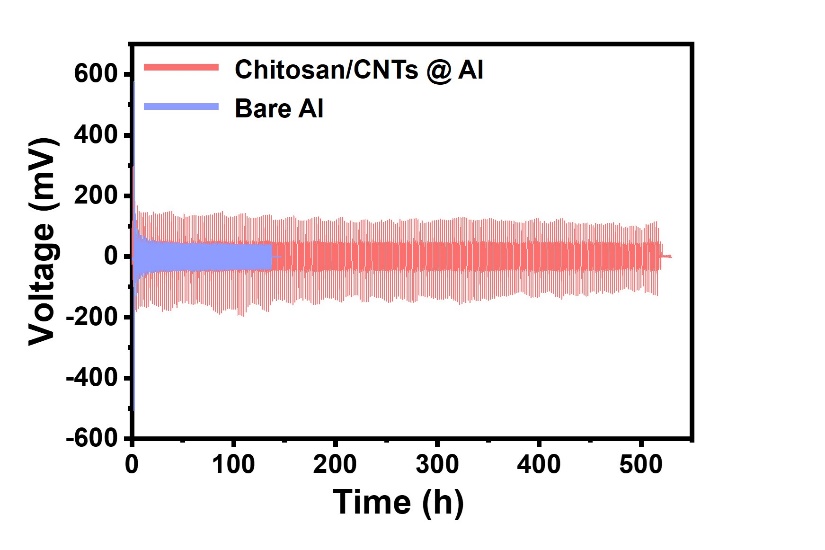


**Fig. S32** Cycling performance of bare Al and chitosan/CNTs @ Al symmetric batteries at 0.1 mA cm^-2^,0.1 mAh cm^-2^


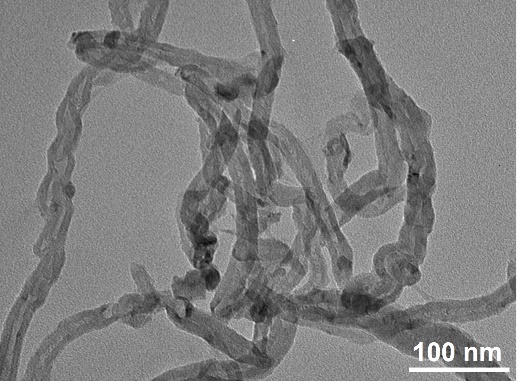


**Fig. S33** TEM of commercial CNTs

**Table S1** Comparison in electrochemical performance of Zn//Zn symmetric batteries with different anodes

| Modified strategies | Current density, (mA cm^-2^) | Areal capacity,  (mAh cm^-2^) | Lifespan  (hours) | Refs. |
| --- | --- | --- | --- | --- |
| Zn@Zn-BTC | 1 | 1 | 800 | [S5] |
| Zn@ZrP | 0.5 | 1 | 780 | [S6] |
| PZIL | 1 | 1 | 1000 | [S7] |
| Sb_2_O_3_@Zn | 1 | 0.5 | 1000 | [S8] |
| Zn@Sb | 1 | 1 | 800 | [S9] |
| CeO_2_@Zn | 0.5 | 0.25 | 1300 | [S10] |
| PDA@Zn | 1 | 1 | 1100 | [S11] |
| EDTA-2Na | 5 | 2.5 | 500 | [S12] |
| ZP-coated | 5 | 2.5 | 250 | [S13] |
| IF-BTO | 0.25 | 0.25 | 1600 | [S14] |
| Triple-gradient | 5 | 2.5 | 400 | [S15] |
| Cu@CNFs | 2.5 | 2.5 | 800 | [S16] |
| F-GQDs | 0.5 | 0.25 | 1800 | [S17] |
| MB @ Zn | 2 | 1 | 650 | [S18] |
| SC-PPS@Zn | 1 | 1 | 600 | [S19] |
| **Chitosan/CNTs @ Zn** | **2** | **1** | **2070** | **This Work** |
|  | **5** | **2.5** | **870** |  |

**Table S2** Main parameters used in the simulation of the battery packs

| Parameter | Bare Zn // V_2_O_5_ | Chitosan/CNTs @ Zn // V_2_O_5_ |
| --- | --- | --- |
| Positive electrode active material specific capacity (mAh·g^-1^) | 362 | 418 |
| Positive electrode active material density (g·cm^-3^) | 3.36 | 3.36 |
| Positive electrode composition (AM:CC:B, weight fractions) | 94:3:3 | 94:3:3 |
| Positive electrode active material cost  ($·kg^-1^) | 10 | 10 |
| Positive electrode porosity | 25% | 25% |
| Positive electrode current collector thickness (µm) | 20 (Stainless steel) | 20 (Stainless steel) |
| Positive electrode current collector cost  ($·m^-2^) | 0.8 | 0.8 |
| Negative electrode active material specific capacity (mAh·g^-1^) | 820 | 820 |
| Negative electrode active material density (g·cm^-3^) | 7.14 | 7.14 |
| Negative electrode composition (AM:CC:B, weight fractions) | 100:0:0 | 100:0:0 |
| Negative electrode active material cost  ($·kg^-1^) | 2.50 | 2.50 |
| Negative electrode current collector thickness (µm) | 20 (Stainless steel) | 20 (Stainless steel) |
| Negative electrode current collector cost ($·m^-2^) | 0.8 | 0.8 |
| Electrolyte density (g·cm^-3^) | 1.28 | 1.28 |
| Electrolyte cost ($·L^-1^) | 1 | 1 |
| Separator thickness (µm) | 15 | 15 |
| N/P ratio^a^ | 1.1 | 1.1 |

^a^ Ratio between the capacity of the anode and the capacity of the cathode

Table S3 Main results of the simulation of the battery packs

| Parameter | Bare Zn // V_2_O_5_ | Chitosan/CNTs @ Zn // V_2_O_5_ |
| --- | --- | --- |
| Positive electrode active material cost ($·pack^-1^) | 572 | 506 |
| Negative electrode active material cost ($·pack^-1^) | 68 | 70 |
| Electrodes preparation cost ($·pack^-1^) | 123 | 109 |
| Positive electrode current collector cost ($·pack^-1^) | 98 | 87 |
| Negative electrode current collector cost ($·pack^-1^) | 102 | 91 |
| Separators cost ($·pack^-1^) | 24 | 22 |
| Electrolyte cost ($·pack^-1^) | 8 | 7 |
| Battery system total energy (kWh) | 11.5 | 11.5 |
| Battery system rated power (KW) | 1 | 1 |
| Battery system capacity, (Ah) | 121 | 123 |
| Battery system nominal operating voltage (V) | 95 | 93 |
| Positive electrode thickness (µm) | 120 | 120 |
| Negative electrode thickness (µm) | 18 | 21 |
| Cell volume (L) | 0.37 | 0.34 |
| Cell mass (kg) | 0.73 | 0.68 |
| Cell capacity (Ah) | 121 | 123 |
| Cell specific energy (Wh·kg^-1^) | 109 | 117 |
| Cell energy density (Wh·L^-1^) | 216 | 235 |
| Cell cost ($·kWh^-1^) | 162 | 151 |

Table S4 Detailed data of the simulation of the battery packs

|  | Bare Zn // V_2_O_5_ | Chitosan/CNTs @ Zn // V_2_O_5_ |
| --- | --- | --- |
| Total cost of cell materials ($/cell) | 7.48 | 7.11 |
| Direct labor ($/cell) | 0.87 | 0.85 |
| Variable overhead  ($/cell) | 0.72 | 0.70 |
| Total fixed costs ($/cell) | 3.42 | 3.35 |
| Total cost of materials ($/year) | 28,222,881 | 25,605,692 |
| Total other variable costs ($/year) | 5,719,323 | 5,594,047 |
| Total fixed costs  ($/year) | 12,313,132 | 12,067,922 |
| Total year cost ($/year) | 46,255,336 | 43,267,661 |
| Energy density (Wh/l) | 216 | 235 |
| Specific energy (Wh/kg) | 109 | 117 |

**Supplementary References**

1. T. Lu, F. Chen, Multiwfn: a multifunctional wavefunction analyzer. J. Comput. Chem. **33**(5), 580–592 (2012). <https://doi.org/10.1002/jcc.22885>
2. S. A. P. A. Nelson, K. G. Gallagher, D. W. Dees. *Modeling the performance and cost of lithium-ion batteries for electric-drive vehicles*. (Argonne National Laboratory; USA, IL, Lemont, **2019**).
3. V_2_O_5_ vanadium pentoxide flake 98% price usd/lb, https://vanadiumprice.com/. Accessed 4 January 2025
4. A. Innocenti, D. Bresser, J. Garche, S. Passerini, A critical discussion of the current availability of lithium and zinc for use in batteries. Nat. Commun. **15**, 4068 (2024). <https://doi.org/10.1038/s41467-024-48368-0>
5. Y. Wang, Y. Liu, H. Wang, S. Dou, W. Gan et al., MOF-based ionic sieve interphase for regulated Zn^2+^ flux toward dendrite-free aqueous zinc-ion batteries. J. Mater. Chem. A **10**(8), 4366–4375 (2022). <https://doi.org/10.1039/D1TA10245A>
6. J. Yan, M. Ye, Y. Zhang, Y. Tang, C.L. Cheng, Layered zirconium phosphate-based artificial solid electrolyte interface with zinc ion channels towards dendrite-free Zn metal anodes. Chem. Eng. J. **432**, 134227 (2022). <https://doi.org/10.1016/j.cej.2021.134227>
7. Z. Meng, Y. Jiao, P. Wu, Alleviating side reactions on Zn anodes for aqueous batteries by a cell membrane derived phosphorylcholine zwitterionic protective layer. Angew. Chem. Int. Ed. **62**(31), e202307271 (2023). <https://doi.org/10.1002/anie.202307271>
8. P. Xiao, Y. Wu, K. Liu, X. Feng, J. Liang et al., An ultrathin inorganic molecular crystal interfacial layer for stable Zn anode. Angew. Chem. Int. Ed. **62**(40), e202309765 (2023). <https://doi.org/10.1002/anie.202309765>
9. L. Hong, L.-Y. Wang, Y. Wang, X. Wu, W. Huang et al., Toward hydrogen-free and dendrite-free aqueous zinc batteries: formation of zincophilic protective layer on Zn anodes. Adv. Sci. **9**(6), 2104866 (2022). <https://doi.org/10.1002/advs.202104866>
10. H. Liu, J.-G. Wang, W. Hua, H. Sun, H. Yu et al., Building ohmic contact interfaces toward ultrastable Zn metal anodes. Adv. Sci. **8**(23), e2102612 (2021). <https://doi.org/10.1002/advs.202102612>
11. X. Zeng, K. Xie, S. Liu, S. Zhang, J. Hao et al., Bio-inspired design of an *in situ* multifunctional polymeric solid–electrolyte interphase for Zn metal anode cycling at 30 mA cm^−2^ and 30 mA h cm^−2^. Energy Environ. Sci. **14**(11), 5947–5957 (2021). <https://doi.org/10.1039/D1EE01851E>
12. J. Cao, D. Zhang, R. Chanajaree, Y. Yue, Z. Zeng et al., Stabilizing zinc anode *via* a chelation and desolvation electrolyte additive. Adv. Powder Mater. **1**(1), 100007 (2022). <https://doi.org/10.1016/j.apmate.2021.09.007>
13. H.J. Kim, S. Kim, K. Heo, J.-H. Lim, H. Yashiro et al., Nature of zinc-derived dendrite and its suppression in mildly acidic aqueous zinc-ion battery. Adv. Energy Mater. **13**(2), 2203189 (2023). <https://doi.org/10.1002/aenm.202203189>
14. S. Zhou, X. Meng, C. Fu, D. Xu, J. Li et al., Aligned dipoles induced electric-field promoting zinc-ion de-solvation toward highly stable dendrite-free zinc-metal batteries (small 49/2023). Small **19**(49), 2370407 (2023). <https://doi.org/10.1002/smll.202370417>
15. Y. Gao, Q. Cao, J. Pu, X. Zhao, G. Fu et al., Stable Zn anodes with triple gradients. Adv. Mater. **35**(6), 2207573 (2023). https://doi.org/10.1002/adma.202207573
16. S. Yang, Y. Li, H. Du, Y. Liu, Y. Xiang et al., Copper nanoparticle-modified carbon nanofiber for seeded zinc deposition enables stable Zn metal anode. ACS Sustainable Chem. Eng. **10**(38), 12630–12641 (2022). <https://doi.org/10.1021/acssuschemeng.2c03328>
17. W. Han, H. Lee, Y. Liu, Y. Kim, H. Chu et al., Toward highly reversible aqueous zinc-ion batteries: nanoscale-regulated zinc nucleation *via* graphene quantum dots functionalized with multiple functional groups. Chem. Eng. J. **452**, 139090 (2023). <https://doi.org/10.1016/j.cej.2022.139090>
18. T. Huang, K. Xu, N. Jia, L. Yang, H. Liu et al., Intrinsic interfacial dynamic engineering of zincophilic microbrushes *via* regulating Zn deposition for highly reversible aqueous zinc ion battery. Adv. Mater. **35**(5), 2205206 (2023). <https://doi.org/10.1002/adma.202205206>
19. L. Zhang, J. Huang, H. Guo, L. Ge, Z. Tian et al., Tuning ion transport at the anode-electrolyte interface *via* a sulfonate-rich ion-exchange layer for durable zinc-iodine batteries. Adv. Energy Mater. **13**(13), 2203790 (2023). <https://doi.org/10.1002/aenm.202203790>
